# The efficacy of dietary therapies in modulating inflammatory biomarkers, clinical remission and quality of life in patients with inflammatory bowel disease: a network meta-analysis of 15 interventions

**DOI:** 10.3389/fnut.2025.1668590

**Published:** 2025-10-06

**Authors:** Kexi Wei, Min Li, Yuqing Zhao

**Affiliations:** ^1^Affiliated Hospital of North Sichuan Medical College, Nanchong, China; ^2^North Sichuan Medical College, Nanchong, China

**Keywords:** dietary patterns, inflammatory bowel disease, Crohn’s disease, ulcerative colitis, randomized, controlled trials, network meta-analysis

## Abstract

**Background:**

Scientific dietary interventions are useful methods for managing inflammatory bowel disease. It is unclear which dietary pattern is most effective in improving IBD symptoms. Therefore, this network meta-analysis compared the impact of popular dietary patterns on patients with established IBD.

**Methods:**

A computerized search of randomized controlled trials on the use of dietary therapy to improve inflammatory bowel disease in both Chinese and English databases. The primary outcome measures were CRP, ALB, IBDQ and MES. Stata 16.0 software was used for the network meta-analysis.

**Results:**

A total of 25 RCTs were ultimately included. The study included the following 15 treatments. The network meta-analysis revealed that, for reducing CRP levels, LFD + EN was significantly more effective than LRD [MD = −5.21 mg/L, 95% CI (−7.05, −3.36)], RD [MD = −4.63 mg/L, 95% CI (−6.22, −3.03)], CDED + EN [MD = −4.48 mg/L, 95% CI (−7.45, −1.51)], LFD [MD = −4.47 mg/L, 95% CI (−6.27, −2.67)], MD + LFD + EN [MD = −3.68 mg/L, 95% CI (−5.90, −1.45)] and EN [MD = −1.26 mg/L, 95% CI (−2.29, −0.22)]. Conversely, LFD + EN was also superior in increasing ALB levels when compared to EN [MD = 3.64 g/L, 95% CI (0.71, 6.57)], LFD [MD = 6.35 g/L, 95% CI (2.85, 9.84)], RD [MD = 6.40 g/L, 95% CI (3.25, 9.54)], LRD [MD = 6.34 g/L, 95% CI (2.83, 9.84)], MD [MD = 6.34 g/L, 95% CI (2.83, 9.84)], CDED + EN [MD = 8.40 g/L, 95% CI (4.18, 12.61)] and lgG-ED [MD = 8.73 g/L, 95% CI (4.34, 13.11)]. Regarding MES, lgG-ED [SMD = 1.07, 95% CI (0.64, 1.50)], LFD [SMD = 0.75, 95% CI (0.48, 1.03)], EN [SMD = 0.64, 95% CI (0.27, 1.01)] all demonstrated a significant reduction in scores compared to RD. No significant difference was found in IBDQ.

**Conclusion:**

For reducing systemic inflammation (CRP, ALB), LFD + EN was ranked as the most effective strategy. For improving quality of life (IBDQ), MD + LFD + EN showed the highest probability of being the best. For inducing endoscopic remission (MES), IgG-ED was ranked highest among the compared interventions. In the future, evidence-based dietary interventions could be used in clinical practice.

**Systematic review registration:**

https://www.crd.york.ac.uk/PROSPERO/view/CRD420251038185.

## 1 Introduction

Inflammatory bowel disease (IBD), comprising Crohn’s disease (CD) and ulcerative colitis (UC), represents a group of chronic, idiopathic inflammatory disorders of the gastrointestinal tract (1). The hallmark clinical manifestations include chronic diarrhea, abdominal pain, hematochezia, and mucus discharge in stools. IBD is characterized by a relapsing–remitting disease course with frequent recurrence (2). The global prevalence of IBD has markedly increased in recent decades, affecting both developed and developing countries (3). Emerging evidence strongly implicates dietary modifications, lifestyle changes, and industrialization as key environmental factors contributing to IBD pathogenesis. Despite therapeutic advances, including biologics and immunomodulators, a substantial proportion of patients still face challenges such as suboptimal response, adverse drug reactions, and high treatment costs. Consequently, the investigation of accessible and safe nonpharmacological interventions, particularly dietary modifications, has become an important research priority.

Multiple hypotheses have been proposed to explain diet-IBD interactions. These include diet-induced alterations in gut microbiota composition, changes in microbial metabolite profiles, modifications of mucosal immunity, and disruption of the intestinal epithelial barrier. However, the exact pathophysiological mechanisms remain to be fully elucidated (4). The increasing incidence of IBD has been strongly associated with substantial changes in dietary patterns during recent decades. Accumulating evidence has demonstrated a significant association between dietary factors and IBD development (5). Dietary factors may influence IBD pathogenesis through two distinct mechanisms: direct host effects and indirect effects mediated through the modulation of the gut microbiota composition and function (6). Diet serves as a primary determinant of the composition of the gut microbiota, and dietary modifications can significantly alter the microbial community structure (7). Furthermore, dietary components influence inflammatory cells and mucosal defenses directly, as well as microbial morphology and metabolism, to regulate immune and inflammatory pathways (8, 9). Therefore, investigating dietary factors as modifiable determinants of IBD risk and evaluating nutritional interventions as potential primary or adjunctive therapies for IBD have gained significant research attention.

Conventional systematic reviews and meta-analyses have primarily focused on individual dietary interventions (e.g., enteral nutrition versus standard diet), limiting their ability to compare the effectiveness of multiple nutritional approaches. Numerous studies have investigated nutritional therapies for IBD, many demonstrating advantages over conventional care. However, the current evidence remains insufficient to establish a hierarchy of therapeutic efficacy among these dietary interventions. Therefore, to address this critical evidence gap, we conducted this network meta-analysis to answer the following key question: What is the comparative efficacy and safety of various dietary interventions for improving outcomes in patients with inflammatory bowel disease?

## 2 Methods

### 2.1 Study design and registration

We designed and wrote this paper according to the Preferred Reporting Items for Systematic Reviews and Meta-Analyses (PRISMA) 2020 statement (10) and registered the protocol with PROSPERO (CRD420251038185).

### 2.2 Inclusion and exclusion criteria

Studies meeting the following criteria were included in this research: (1) patients meeting the diagnostic criteria for IBD as defined by the 2019 European Crohn’s and Colitis Organization (ECCO) guidelines (11) and the 2018 Chinese Medical Association (CMA) guidelines (2), including both CD and UC cases. (2) Participants in the intervention group received various dietary interventions. (3) The control group received another dietary pattern or a regular diet. (Detailed dietary protocols are provided in the Supplementary Table 2). (4) Primary outcomes included at least one of the following measures: (a) C-reactive protein (CRP), (b) albumin (ALB), (c) the Inflammatory Bowel Disease Questionnaire (IBDQ), (d) the Mayo Endoscopic Score (MES) (5) Study type: randomized controlled trial (RCT).

The exclusion criteria were as follows: (1) Non-English or non-Chinese publications. (2) Duplicate publications. (3) Unavailable full-text articles or insufficient data for extraction. (4) Nonrandomized study designs: animal studies, meta-analyses/reviews, conference abstracts, letters/responses to editors, guidelines, or case reports. (5) Studies without dietary intervention components.

Two reviewers (KW and YZ) independently screened the records, with disagreements resolved by consensus with the third reviewer (ML).

### 2.3 Search strategy

A computerized search of randomized controlled trials on the use of dietary therapy for IBD in PubMed, Embase, the Cochrane Library, Web of Science, CNKI, WanFang Data, VIP and SinoMed was conducted from inception to March 31, 2025. A systematic search strategy was developed using both controlled vocabulary (e.g., MeSH terms) and free-text terms, with database-specific adaptations to account for variations in indexing systems and search functionalities. Additionally, we searched reference lists from published relevant systematic reviews and meta-analyses. The search is detailed in Supplementary Table 1.

### 2.4 Data extraction and synthesis

Two investigators (KW and YZ) independently screened the literature and extracted the data after importing all the retrieved records into EndNote20 and removing duplicates. A standardized Excel spreadsheet containing the following variables was developed for data extraction: first author, publication year, sample size, mean age, disease duration, intervention duration, diagnostic standards, intervention characteristics, and outcomes. Following independent data extraction by two investigators (KW and YZ), discrepancies were resolved through consensus discussion with referrals to the original publications when necessary. The method and formula for the three-armed experiment to combine two interventions are shown in the Supplemental Formula 1.

### 2.5 Quality assessment

Risk of bias in the included studies was assessed using the Cochrane risk of bias assessment tool (RoB2) (12) for five domains—allocation concealment, intervention (participants and investigators) blinding, missing outcome data, outcome measurement and reporting, randomization process, and selection of reported results. Each domain received a bias assessment of “low risk,” “high risk,” or “some concerns.” Two independent reviewers (KW and YZ) conducted quality assessment, with discrepancies resolved through consultation with a third researcher (ML) to establish consensus.

### 2.6 Statistical analysis

The standardized mean difference (SMD) was used to analyze the IBDQ and MES outcomes, whereas the mean difference (MD) was used for the CRP and ALB measurements. All pooled effect estimates were quantified via point estimates with corresponding 95% confidence intervals (CIs).

This study employed the mvmeta package in Stata 16.0 and Stata 12.0 software for network and routine meta-analyses, and heatmaps were generated with R 4.3.1. Node sizes in the network plots were proportionate to the overall sample size for each intervention, and line thickness correlated with the number of direct comparisons across interventions. Inconsistency models were used to evaluate global consistency, and node-splitting analysis was used to look for local inconsistencies when they were found. We evaluated consistency by performing node-splitting to explore direct and indirect evidence comparison and a *p* value for inconsistency. Treatment rankings were evaluated via surface under the cumulative ranking curve (SUCRA), where higher values (0–100%) indicate a greater likelihood of being the optimal intervention.

To investigate possible sources of heterogeneity, predefined subgroup analyses stratified by disease duration (≤6 weeks vs. > 6 weeks) and disease subtype (CD, UC, and mixed IBD) were conducted. The *I*^2^ statistic was used to estimate heterogeneity: low heterogeneity was defined as *I*^2^ values <25%, moderate heterogeneity as *I*^2^ values 25–75%, and high heterogeneity as *I*^2^ values >75%. The robustness of the findings was assessed by sensitivity analysis using random effects models.

## 3 Results

### 3.1 Literature search and screening process

**Figure 1:**
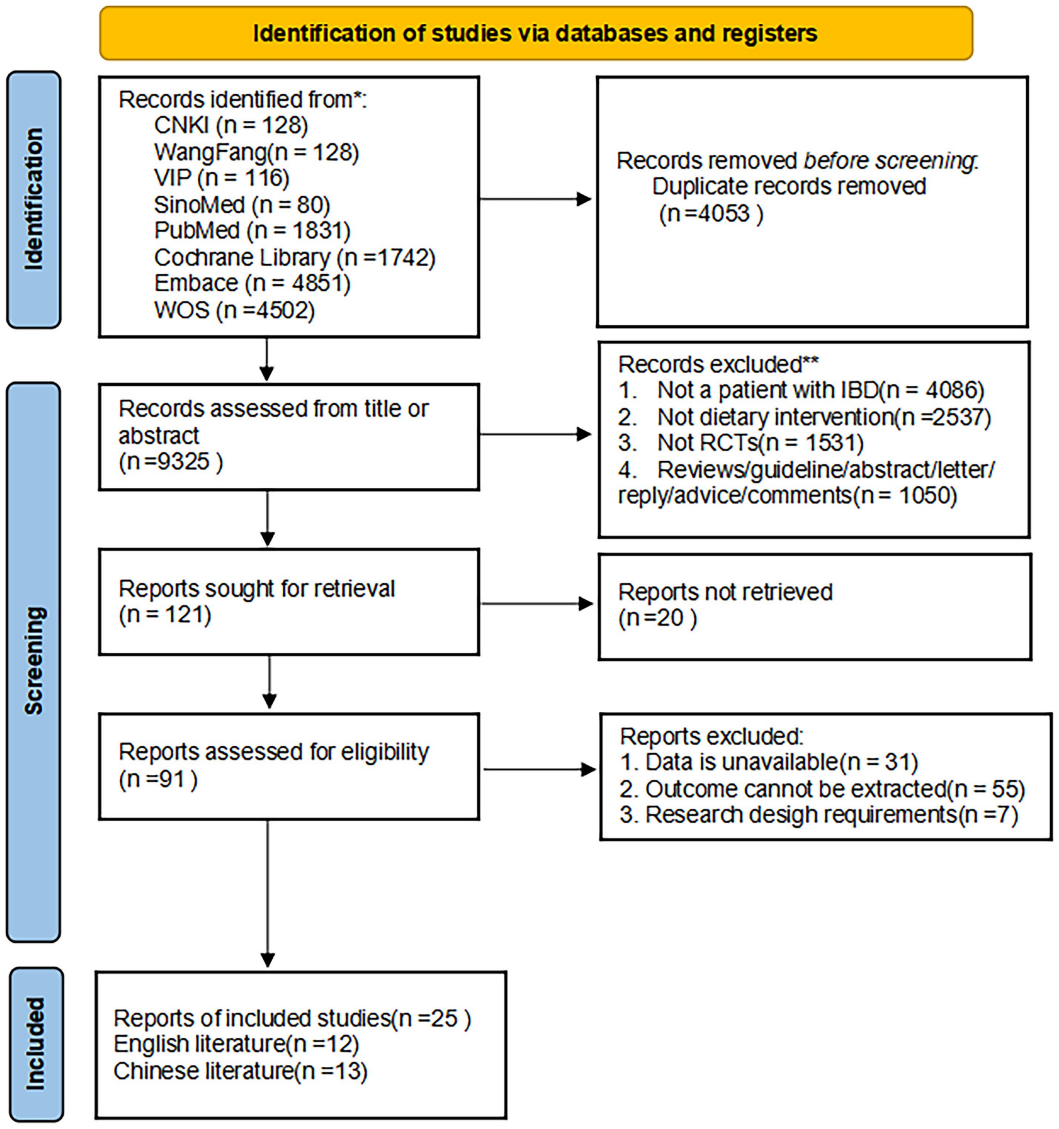
The PRISMA flowchart of the literature search and selection. This flow diagram shows the process used to identify relevant records for the network meta-analysis. The systematic literature search was performed from database inception to March 31, 2025. RCT, randomized controlled trial.

The initial database search identified 13,378 potentially relevant records, through which 25 RCTs (13–37) were ultimately included (13 Chinese-language (13, 14, 16, 17, 20, 24, 32–37) and 12 English-language (15, 19, 21–23, 25–31) publications) after rigorous multistage screening. Of these, collectively involving 1,829 cases, including 930 cases in the experimental group and 899 cases in the control group. The PRISMA flow diagram for research selection is displayed in Figure 1.

### 3.2 Basic characteristics of the included studies

The final analysis included 25 eligible RCTs comprising a total of 1,885 participants. Fifteen distinct dietary interventions were evaluated: Regular diet (RD), high-FODMAP diet (HFD), Mediterranean diet (MD), low-residue diet (LRD), enteral nutrition (EN), specific carbohydrate diet (SCD), Crohn’s disease exclusion diet (CDED), low-FODMAP diet (LFD), IgG-guided exclusion diet (IgG-ED), high-fiber food (HFF), low-FODMAP diet + enteral nutrition, Crohn’s disease exclusion diet + enteral nutrition, Mediterranean diet + low-FODMAP diet + enteral nutrition, Canada’s Food Guide (CFG), and anti-inflammatory diet (AID). The study designs consisted of 2 three-arm trials and 23 two-arm parallel-group trials. Table 1 summarizes the key characteristics of the included studies.

**Table 1:** Characteristics of the included studies.

Author (year)	Sample size (*n*)		Sex (male)		Age (years)		Disease course (years)		Intervention time		Diagnostic standards	Intervention characteristics		Out-comes
	T	C	T	C	T	C	T	C	T	C		Treatment	Control	
Weiguang et al. (13) (2021)	63	66	—	—	44.53 ± 11.59	45.32 ± 11.73	8.71 ± 2.84	8.14 ± 3.27	6 weeks	6 weeks	Consensus Opinion on Integrative Chinese and Western Medicine for the Treatment of Ulcerative Colitis (2017)	LFD	RD	②
Mei et al. (14) (2024)	68	66	38	31	40.18 ± 5.12	39.77 ± 6.22	3.14 ± 1.23	3.36 ± 0.95	12 weeks	12 weeks	Chinese guidelines for the diagnosis and treatment of ulcerative colitis (2023)	LFD	RD	④
Narimani et al. (15) (2024)	25	21	10	13	34.88 ± 9.53	39.76 ± 12.46	5.53 ± 7.76	5.71 ± 6.17	6 weeks	6 weeks	Diagnosed as UC	MD + LFD + EN	RD	①③
Yan et al. (16) (2021)	65	32	29	18	48.12 ± 9.76	45.28 ± 10.16	8.51 ± 2.87	8. 2 ± 3. 3	6 weeks	6 weeks	Diagnostic criteria for UC developed by the Chinese Society of Gastroenterology of the Chinese Medical Association (2018)	LFD	RD	①②④
Li et al. (17) (2023)	50	50	32	34	46.92 ± 3.21	46.89 ± 3.24	—	—	6 weeks	6 weeks	Diagnostic criteria for UC developed by the Chinese Society of Gastroenterology of the Chinese Medical Association (2007)	EN	RD	②
Ailing (18) (2024)	31	31	21	20	37.70 ± 8.59	37.59 ± 8.45	5.42 ± 2.23	5.32 ± 2.15	2 months	2 months	Diagnosed as IBD	EN	RD	①
Jian et al. (19) (2018)	49	48	25	22	38 ± 11	39 ± 12	—	—	6 months	6 months	Diagnosed as UC	IgG-ED	RD	②④
Jingke et al. (20) (2022)	54	53	35	32	8.35 ± 1.29	8.30 ± 1.63	1.59 ± 0.28	1.67 ± 0.26	6 weeks	6 weeks	Diagnosed as UC	LFD + EN	EN	①②
Suskind et al. (21) (2020)	8	2	—	—	—	—	—	—	12 weeks	12 weeks	Diagnosed as CD	SCD	RD	
Arcucci et al. (22) (2024)	11	10	—	—	10.65 ± 7.39	13.59 ± 1.3	—	—	12 weeks	12 weeks	ESPGHAN revised porto criteria for the diagnosis of inflammatory bowel disease in children and adolescents	CDED + EN	RD	①②
Yanai et al. (23) (2020)	15	24	—	—	13.8 ± 2.8	14.5 ± 2.6	2.4 ± 6	2 ± 4.8	6 weeks	6 weeks	—	CDED + EN	CDED	
Haiqin (24) (2018)	30	30	17	18	38 ± 7	39 ± 6	19 ± 7 (d)	18 ± 7 (d)	4 weeks	4 weeks	AGA Crohn’s Disease Treatment Guidelines	HFF	RD	③
Keshteli et al. (25) (2022)	25	24	—	—	—	—	—	—	6 months	6 months	—	AID	RD	③
Marsh et al. (26) (2024)	26	26	—	—	—	—	—	—	6 months	6 months	—	AID	CFG	③
Takagi et al. (27) (2009)	26	25	—	—	30.8 ± 11.1	28.9 ± 8.1	4.1 ± 4.2	5.6 ± 6.5	13 months	13 months	Diagnosed as CD	EN	RD	③
Cox et al. (28) (2020)	27	25	10	13	33 ± 11	40 ± 13	7 ± 8	11 ± 11	4 weeks	4 weeks	—	LFD	RD	①③
El Amrousy et al. (29) (2022)	50	50	28	26	13.8 ± 2.6	13.1 ± 1.9	3.1 ± 1.6	3.4 ± 1.3	12 weeks	12 weeks	Diagnosed as CD or UC	MD	RD	①②
Tapete et al. (30) (2019)	25	25	—	—	43.9 ± 17		—	—	8 weeks	8 weeks	Diagnosed as CD or UC	LFD	HFD	③
Bodini et al. (31) (2019)	26	29	7	17	41 ± 3.5	47 ± 3.25	—	—	6 weeks	6 weeks	Diagnosed as IBD	LFD	RD	①③
Qianhong and Jiaqi (32) (2013)	51	55	31	29	42.2 ± 14.6	43.5 ± 15.4	5.3 ± 0.4		20 days	20 days	—	EN	LRD	①②
Ji et al. (33) (2024)	58	58	—	—	45.85 ± 7.32	43.31 ± 10.04	—	—	6 weeks	6 weeks	Diagnosed as UC	EN	RD	①④
Shubo and Jing (34) (2018)	24	24	—	—	35 ± 13.7	36 ± 14.8	—	—	1 months	1 months	—	EN	RD	②
Yang et al. (35) (2023)	30	30	17	16	45.62 ± 4.82	45.04 ± 4.52	—	—	—	—	Consensus opinion on the diagnosis and treatment of inflammatory bowel disease (2018—Beijing)	EN	RD	②
Long (36) (2023)	50	50	27	26	46.52 ± 1.36	45.28 ± 1.48	—	—	—	—	Diagnosed as UC	EN	RD	②
Long and Wen (37) (2024)	42	42	22	23	42.13 ± 2.37	41.31 ± 2.32	4.03 ± 1.10	3.91 ± 1.08	3 weeks	3 weeks	Guidance management of ulcerative colitis: summary of NICE guideline updates	EN	LRD	①

T, Treatment group; C, Control group; −, not mentioned. Data are expressed as mean ± SD. RD, regular diet; HFD, high-FODMAP diet; MD, Mediterranean diet; LRD, low-residue diet; EN, enteral nutrition; SCD, specific carbohydrate diet; CDED, Crohn’s disease exclusion diet; LFD, low-FODMAP diet; IgG-ED, IgG-guided exclusion diet; HFF, high-fiber food; CFG, Canada’s Food Guide; AID, anti-inflammatory diet. ① CRP; ② ALB; ③ IBDQ; ④ MES.

### 3.3 Quality assessment

**Figure 2:**
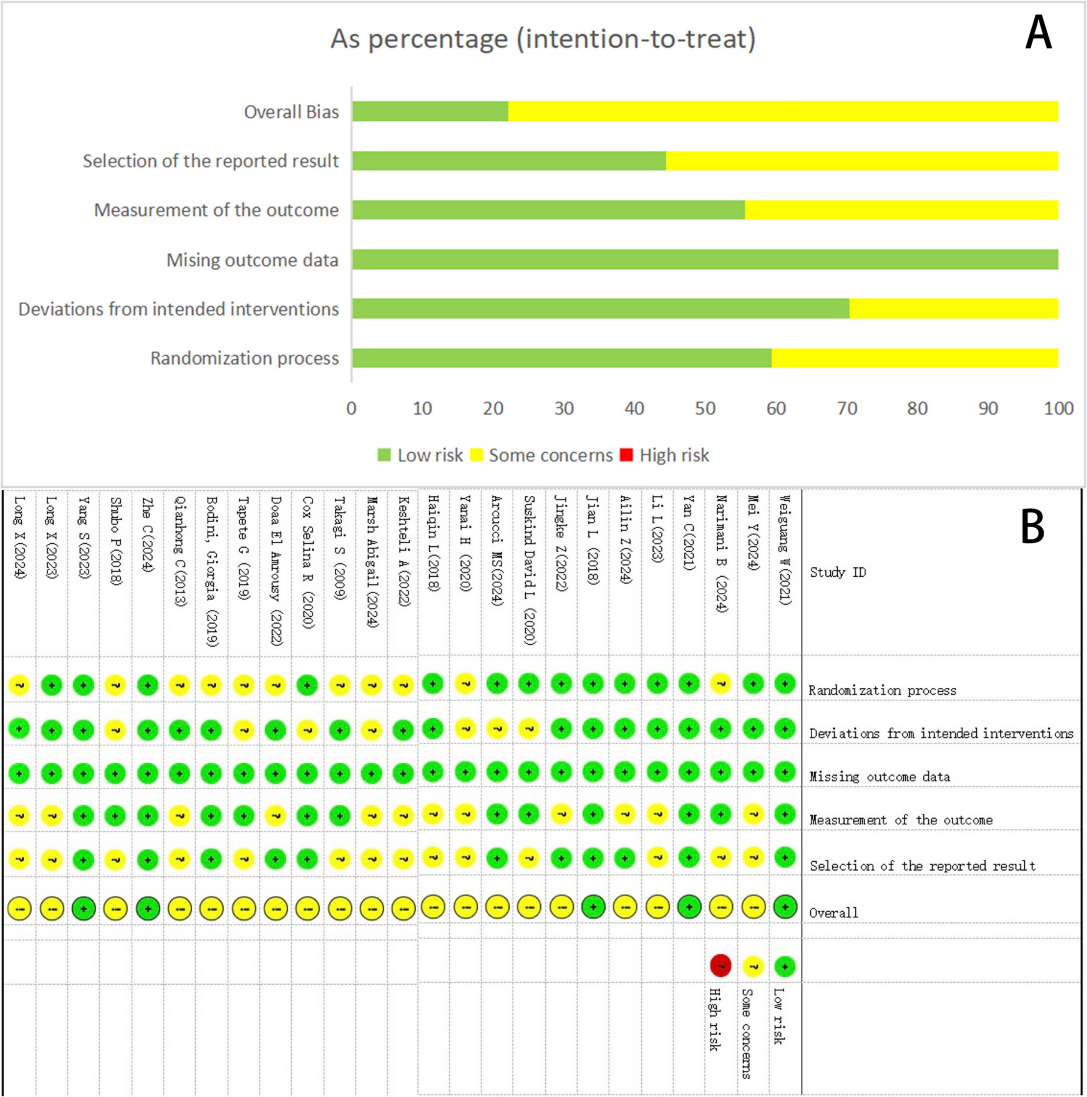
Summary results on the risk of bias (using RoB2) of the included RCTs. **(A)** Percent of studies with categories for risk of bias. **(B)** Summary of the risk of bias in each study.

Among the included studies, all 25 reported baseline group comparability, with 14 studies providing detailed randomization methods (low risk) and 11 studies only briefly mentioning randomization (some concerns). Regarding bias due to deviations from intended interventions, 18 studies demonstrated adequate methods (low risk) versus 7 with insufficient descriptions (some concerns). All studies maintained complete outcome data (low risk of attrition bias). Outcome measurement was considered lower in risk for 13 studies, as it was conducted by third-parties other than the researchers, except for 12 studies, which resulted in limited information (some concerns). And 14 studies were deemed medium risky in the selection of the reported result because of the absence of a prespecified trial protocol (some concerns). No evidence of selective outcome reporting was detected in 11 study (low risk). The results of the risk of bias assessment for the 25 included studies are shown in Figure 2.

### 3.4 Network meta-analysis results

#### 3.4.1 Network evidence diagram

**Figure 3:**
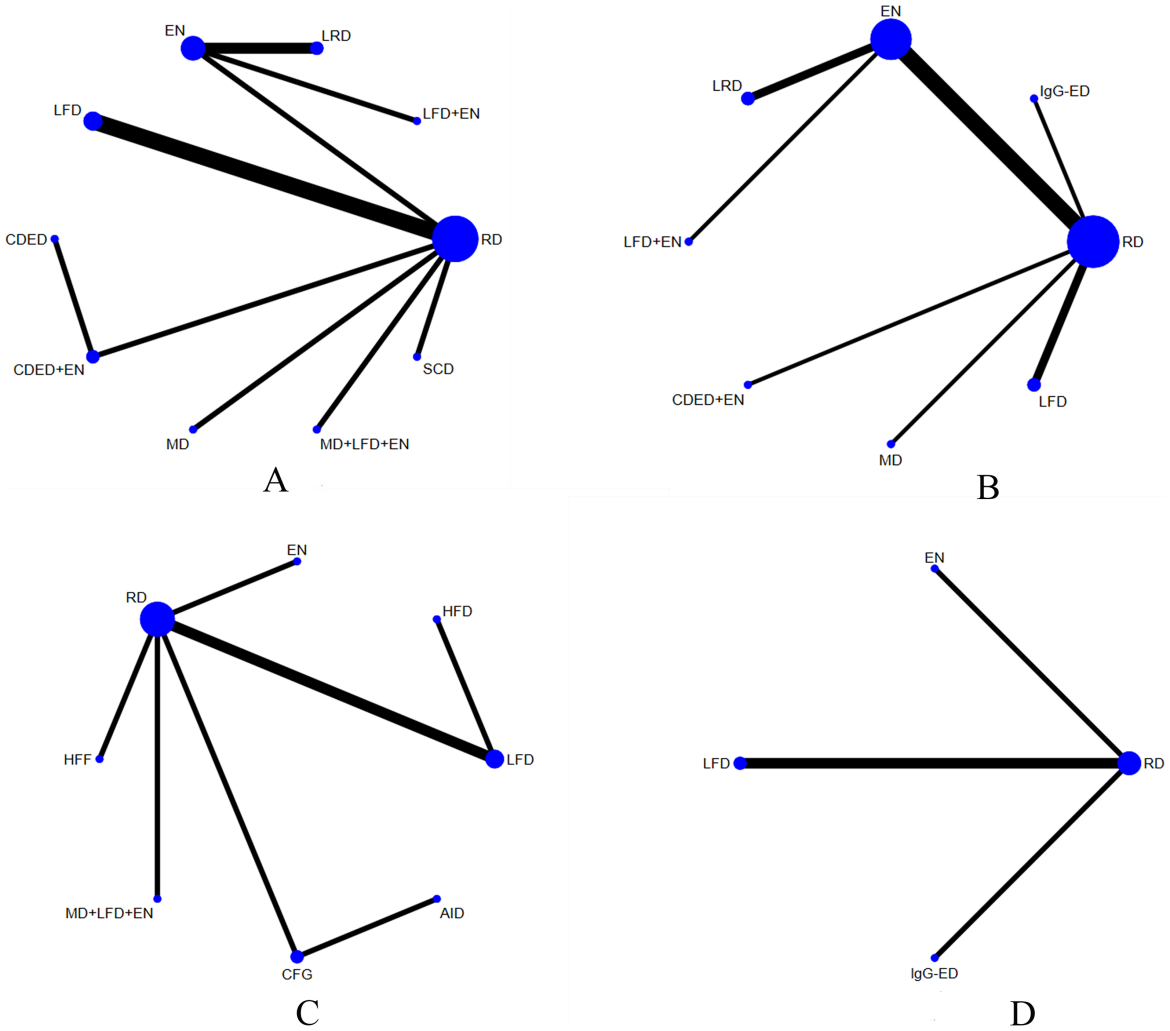
Network plots for CRP **(A)**, ALB **(B)**, IBDQ **(C)**, and the MES **(D)**. The number of participants in that type of intervention is represented by the size of the nodes, and the number of studies used for that comparison is represented by the thickness of the lines connecting the interventions. RD, regular diet; HFD, high-FODMAP diet; MD, Mediterranean diet; LRD, low-residue diet; EN, enteral nutrition; SCD, specific carbohydrate diet; CDED, Crohn’s disease exclusion diet; LFD, low-FODMAP diet; IgG-ED, IgG-guided exclusion diet; HFF, high-fiber food; CFG, Canada’s Food Guide; AID, anti-inflammatory diet.

The 25 included studies covered 15 different interventions. Figure 3 presents the network structure of all competing interventions for each outcome measure. The amount of direct head-to-head comparisons between intervention pairings is correlated with edge thickness, and node diameter is proportional to the total sample size per intervention arm.

#### 3.4.2 CRP

**Figure 4:**
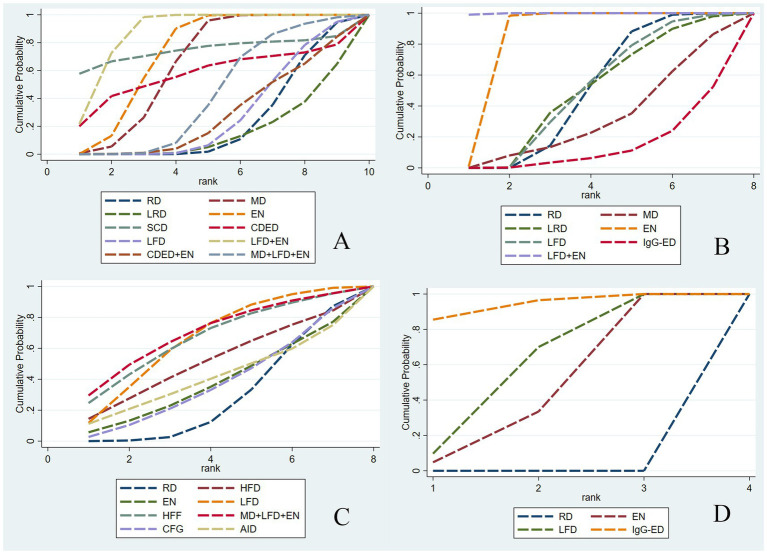
Net meta-analysis ranking results for each outcome indicator. CRP **(A)**, ALB **(B)**, IBDQ **(C)**, and MES **(D)**. RD, regular diet; HFD, high-FODMAP diet; MD, Mediterranean diet; LRD, low-residue diet; EN, enteral nutrition; SCD, specific carbohydrate diet; CDED, Crohn’s disease exclusion diet; LFD, low-FODMAP diet; IgG-ED, IgG-guided exclusion diet; HFF, high-fiber food; CFG, Canada’s Food Guide; AID, anti-inflammatory diet.

**Figure 5:**
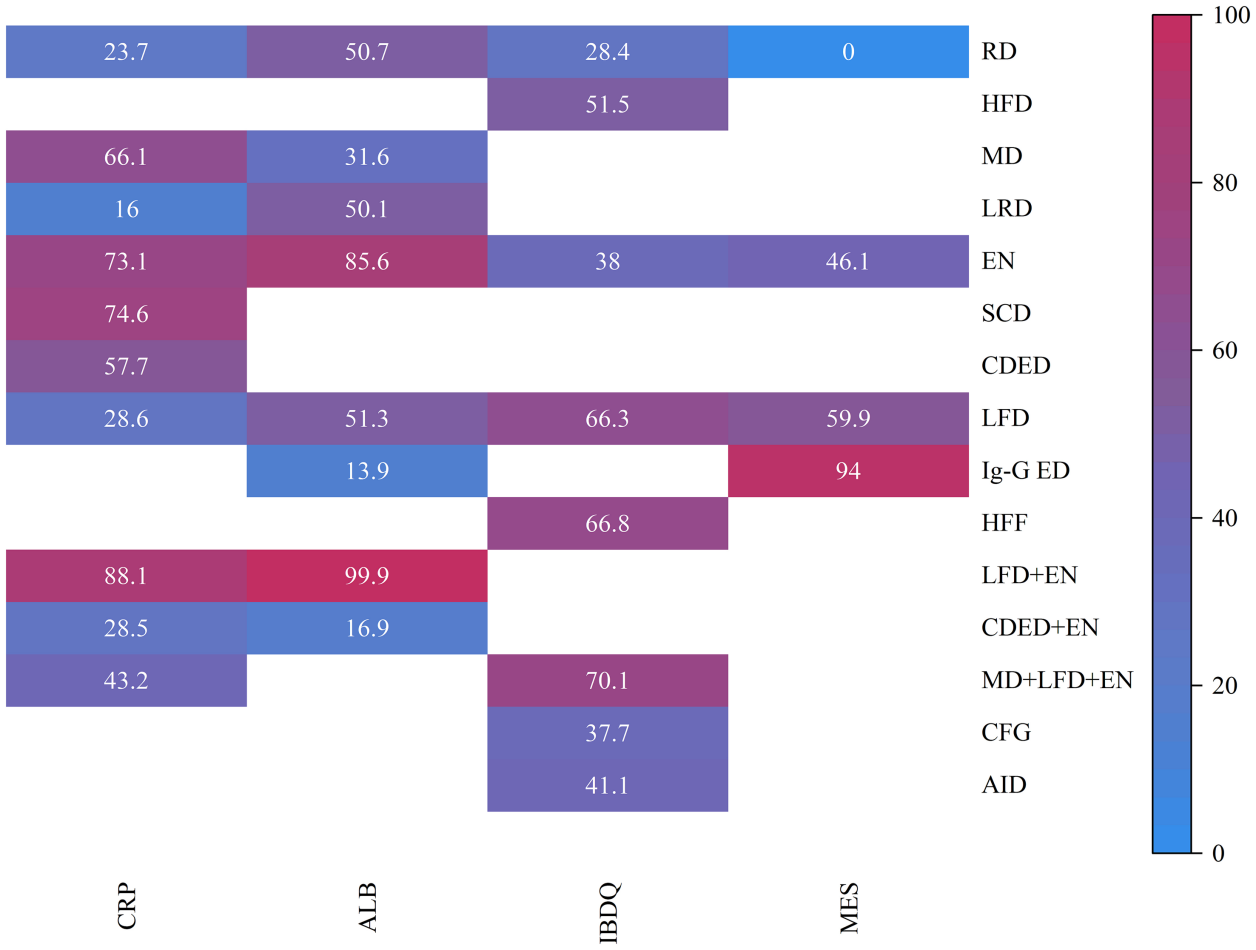
Heat map of SUCRA values. Heat map of SUCRA values for various intervention measures. The SUCRA values, ranging from 0 to 100%, indicate the likelihood of each intervention being the most effective treatment. RD, regular diet; HFD, high-FODMAP diet; MD, Mediterranean diet; LRD, low-residue diet; EN, enteral nutrition; SCD, specific carbohydrate diet; CDED, Crohn’s disease exclusion diet; LFD, low-FODMAP diet; IgG-ED, IgG-guided exclusion diet; HFF, high-fiber food; CFG, Canada’s Food Guide; AID, anti-inflammatory diet.

Draw network diagrams, analyze using a consistency model. Among the 12 included RCTs (15, 16, 18, 20–23, 28, 29, 31, 32, 37) reporting CRP levels, 10 distinct dietary patterns were evaluated. Notably, the network meta-analysis revealed that LFD + EN significantly reduced CRP levels compared with multiple other diets, including LRD [MD = −5.21 mg/L, 95% CI (−7.05, −3.36)], RD [MD = −4.63 mg/L, 95% CI (−6.22, −3.03)], CDED + EN [MD = −4.48 mg/L, 95% CI (−7.45, −1.51)], LFD [MD = −4.47 mg/L, 95% CI (−6.27, −2.67)], MD + LFD + EN [MD = −3.68 mg/L, 95% CI (−5.90, −1.45)] and EN [MD = −1.26 mg/L, 95% CI (−2.29, −0.22)], and the difference was statistically significant (all *p* < 0.05). Furthermore, EN was more effective at lowering CRP levels than LRD, RD, CDED + EN, LFD and MD + LFD + EN (all *p* < 0.05). MD also led to a greater reduction in CRP than three other diets: LRD, RD and LFD (all *p* < 0.05). Statistical significance was not attained by other dietary comparisons (all *p* > 0.05), as detailed in Table 2. SUCRA analysis revealed that LFD + EN (SUCRA = 88.1%) and SCD (SUCRA = 74.6%) were ranked as the most effective interventions for improving CRP (Figure 4A). The complete SUCRA values and intervention rankings for all outcomes are presented in Table 3 and Figure 5.

**Table 2:** Relative effects of different dietary patterns on CRP.

**LFD + EN**									
3.37 (−14.90, 21.65)	**SCD**								
**−1.26 (−2.29, −0.22)**	−4.63 (−22.88, 13.61)	**EN**							
−1.93 (−4.04, 0.18)	−5.30 (−23.56, 12.96)	−0.67 (−2.50, 1.17)	**MD**						
−1.68 (−12.84, 9.48)	−5.05 (−26.34, 16.24)	−0.42 (−11.53, 10.70)	0.25 (−10.88, 11.38)	**CDED**					
**−3.68 (−5.90, −1.45)**	−7.05 (−25.32, 11.22)	**−2.42 (−4.38, −0.45)**	−1.75 (−3.82, 0.33)	−2.00 (−13.15, 9.16)	**MD + LFD + EN**				
**−4.47 (−6.27, −2.67)**	−7.84 (−26.07, 10.38)	**−3.21 (−4.69, −1.73)**	**−2.54 (−4.16, −0.93)**	−2.79 (−13.87, 8.29)	−0.79 (−2.56, 0.97)	**LFD**			
**−4.48 (−7.45, −1.51)**	−7.85 (−26.23, 10.53)	**−3.22 (−6.00, −0.44)**	−2.55 (−5.41, 0.31)	−2.80 (−13.56, 7.96)	−0.80 (−3.75, 2.14)	−0.01 (−2.65, 2.64)	**CDED + EN**		
**−4.63 (−6.22, −3.03)**	−8.00 (−26.20, 10.20)	**−3.37 (−4.58, −2.16)**	**−2.70 (−4.08, −1.32)**	−2.95 (−14.00, 8.10)	−0.95 (−2.50, 0.60)	−0.16 (−1.00, 0.69)	−0.15 (−2.66, 2.36)	**RD**	
**−5.21 (−7.05, −3.36)**	−8.58 (−26.89, 9.73)	**−3.95 (−5.47, −2.42)**	**−3.28 (−5.67, −0.89)**	−3.53 (−14.75, 7.69)	−1.53 (−4.02, 0.96)	−0.74 (−2.86, 1.39)	−0.73 (−3.90, 2.45)	−0.58 (−2.53, 1.37)	**LRD**

The estimates are displayed as the MD and 95% CIs. Table cells are color-coded for interpretation. Dietary interventions are listed in orange-bolded text. Statistically significant results (95% CI excluding zero) are highlighted in dark green and shown in bold. Non-significant results (95% CI including zero) are shown in light green. RD, regular diet; MD, Mediterranean diet; EN, enteral nutrition; SCD, specific carbohydrate diet; CDED, Crohn’s disease exclusion diet; LFD, low-FODMAP diet. LRD, low residue diet.

**Table 3:** Ranking of efficacy of each treatments.

Treatment	CRP		ALB		IBDQ		MES	
	SUCRA/%	Rank	SUCRA/%	Rank	SUCRA/%	Rank	SUCRA/%	Rank
RD	23.7%	9	50.7%	3	28.4%	8	0%	4
HFD	—	—	—	—	51.5%	4	—	—
MD	66.1%	4	31.6%	6	—	—	—	—
LRD	16%	10	50.1%	5	—	—	—	—
EN	73.1%	3	85.6%	2	38%	6	46.1%	3
SCD	74.6%	2	—	—	—	—	—	—
CDED	57.7%	5	—	—	—	—	—	—
LFD	28.6%	7	51.3%	4	66.3%	3	59.9%	2
Ig-G ED	—	—	13.9%	8	—	—	94%	1
HFF	—	—	—	—	66.8%	2	—	—
LFD + EN	88.1%	1	99.9%	1	—	—	—	—
CDED + EN	28.5%	8	16.9%	7	—	—	—	—
MD + LFD + EN	43.2%	6	—	—	70.1%	1	—	—
CFG	—	—	—	—	37.7%	7	—	—
AID	—	—	—	—	41.1%	5	—	—

—, not mentioned. Data are expressed as mean ± SD. RD, regular diet; HFD, high-FODMAP diet; MD, Mediterranean diet; LRD, low-residue diet; EN, enteral nutrition; SCD, specific carbohydrate diet; CDED, Crohn’s disease exclusion diet; LFD, low-FODMAP diet; IgG-ED, IgG-guided exclusion diet; HFF, high-fiber food; CFG, Canada’s Food Guide; AID, anti-inflammatory diet.

#### 3.4.3 ALB

Draw network diagrams, analyze using a consistency model. Among the 12 included RCTs (13, 16, 17, 19, 20, 22, 29, 32–36) reporting ALB levels, 8 distinct dietary patterns were evaluated. Notably, the network meta-analysis revealed that, LFD + EN significantly increased the ALB levels compared with EN [MD = 3.64 g/L, 95% CI (0.71, 6.57)], LFD [MD = 6.35 g/L, 95% CI (2.85, 9.84)], RD [MD = 6.40 g/L, 95% CI (3.25, 9.54)], LRD [MD = 6.34 g/L, 95% CI (2.83, 9.84)], MD [MD = 6.34 g/L, 95% CI (2.83, 9.84)], CDED + EN [MD = 8.40 g/L, 95% CI (4.18, 12.61)] and lgG-ED [MD = 8.73 g/L, 95% CI (4.34, 13.11)], and the differences were statistically significant (all *p* < 0.05). Furthermore, EN intervention produced a greater increase in serum ALB concentration than interventions using LFD, RD, LRD, MD, CDED + EN, or IgG-ED (all *p* < 0.05). The other dietary comparisons did not reach statistical significance (all *p* > 0.05), as detailed in Table 4. SUCRA analysis revealed LFD + EN (SUCRAs: 99.9%) and EN (SUCRAs: 85.6%) as the most effective interventions for improving the ALB concentration (Figure 4B).

**Table 4:** Relative effects of different dietary patterns on ALB.

**LFD + EN**							
**3.64 (0.71, 6.57)**	**EN**						
**6.35 (2.85, 9.84)**	**2.71 (0.80, 4.62)**	**LFD**					
**6.40 (3.25, 9.54)**	**2.76 (1.61, 3.90)**	0.05 (−1.49, 1.58)	**RD**				
**6.34 (2.83, 9.84)**	**2.70 (0.77, 4.63)**	−0.01 (−2.72, 2.70)	−0.06 (−2.29, 2.18)	**LRD**			
**7.40 (3.13, 11.66)**	**3.76 (0.65, 6.86)**	1.05 (−2.22, 4.31)	1.00 (−1.88, 3.88)	1.06 (−2.59, 4.70)	**MD**		
**8.40 (4.18, 12.61)**	**4.76 (1.73, 7.78)**	2.05 (−1.15, 5.24)	2.00 (−0.80, 4.80)	2.06 (−1.53, 5.64)	1.00 (−3.02, 5.02)	**CDED + EN**	
**8.73 (4.34, 13.11)**	**5.09 (1.83, 8.34)**	2.38 (−1.04, 5.79)	2.33 (−0.72, 5.38)	2.39 (−1.40, 6.17)	1.33 (−2.87, 5.53)	0.33 (−3.81, 4.47)	**lgG-ED**

The estimates are displayed as the MD and 95% CIs. Table cells are color-coded for interpretation. Dietary interventions are listed in orange-bolded text. Statistically significant results (95% CI excluding zero) are highlighted in dark green and shown in bold. Non-significant results (95% CI including zero) are shown in light green. RD, regular diet; MD, Mediterranean diet; LRD, low residue diet; EN, enteral nutrition; CDED, Crohn’s disease exclusion diet; LFD, low FODMAP diet; IgG-ED, IgG-guided exclusion diet.

#### 3.4.4 IBDQ

Draw network diagrams, analyze using a consistency model. Among the 8 included RCTs (15, 24–28, 30, 31) reporting IBDQ levels, 8 distinct dietary patterns were evaluated. The network meta-analysis revealed that, compared with RD, all dietary interventions tended to improve IBDQ scores. However, the pairwise comparisons did not yield any statistically significant differences among the dietary regimens (all *p* > 0.05; Table 5). Cumulative probability analysis identified MD + LFD + EN (SUCRA = 70.1%) and HFD (SUCRA = 66.8%) as the most promising interventions for IBDQ improvement (Figure 4C).

**Table 5:** Relative effects of different dietary patterns on IBDQ.

**MD + LFD + EN**							
0.22 (−5.87, 6.30)	**HFF**						
0.47 (−4.80, 5.75)	0.26 (−5.00, 5.51)	**LFD**					
1.25 (−5.53, 8.04)	1.04 (−5.73, 7.81)	0.78 (−3.49, 5.05)	**HFD**				
2.22 (−5.19, 9.63)	2.00 (−5.40, 9.40)	1.74 (−5.01, 8.49)	0.96 (−7.02, 8.95)	**AID**			
2.30 (−3.77, 8.37)	2.08 (−3.97, 8.14)	1.83 (−3.42, 7.07)	1.05 (−5.72, 7.81)	0.08 (−7.31, 7.47)	**EN**		
2.33 (−3.73, 8.40)	2.12 (−3.93, 8.17)	1.86 (−3.38, 7.10)	1.08 (−5.68, 7.84)	0.11 (−4.15, 4.38)	0.03 (−6.00, 6.07)	**CFG**	
2.59 (−1.72, 6.90)	2.38 (−1.91, 6.67)	2.12 (−0.92, 5.16)	1.34 (−3.90, 6.58)	0.37 (−5.65, 6.40)	0.29 (−3.98, 4.57)	0.26 (−4.00, 4.52)	**RD**

The estimates are displayed as the SMD and 95% CIs. Table cells are color-coded for interpretation. Dietary interventions are listed in orange-bolded text. Statistically significant results (95% CI excluding zero) are highlighted in dark green and shown in bold. Non-significant results (95% CI including zero) are shown in light green. RD, regular diet; HFD, high-FODMAP diet; MD, Mediterranean diet; EN, enteral nutrition; LFD, low-FODMAP diet; HFF, high-fiber food; CFG, Canada Food Guide; AID, anti-inflammatory diet.

#### 3.4.5 MES

Draw network diagrams, analyze using a consistency model. Among the 4 included RCTs (14, 16, 19, 20) reporting MES levels, 4 distinct dietary patterns were evaluated. According to the network meta-analysis, lgG-ED [SMD = 1.07, 95% CI (0.64, 1.50)], LFD [SMD = 0.75, 95% CI (0.48, 1.03)], EN [SMD = 0.64, 95% CI (0.27, 1.01)] could considerably lower MES when compared to RD. The differences were statistically significant (all *p* < 0.05). Other dietary comparisons did not reach statistical significance (all *p* > 0.05; Table 6). SUCRA analysis revealed lgG-ED (SUCRAs: 94%) as the most effective interventions for improving the MES concentration (Figure 4D).

**Table 6:** Relative effects of different dietary patterns on MES.

**RD**			
**0.64 (0.27, 1.01)**	**EN**		
**0.75 (0.48, 1.03)**	0.11 (−0.35, 0.58)	**LFD**	
**1.07 (0.64, 1.50)**	0.43 (−0.14, 1.00)	0.32 (−0.19, 0.82)	**IgG-ED**

The estimates are displayed as the SMD and 95% CIs. Table cells are color-coded for interpretation. Dietary interventions are listed in orange-bolded text. Statistically significant results (95% CI excluding zero) are highlighted in dark green and shown in bold. Non-significant results (95% CI including zero) are shown in light green. RD, regular diet; EN, enteral nutrition; IgG-ED, IgG-guided exclusion diet; LFD, low-FODMAP diet.

#### 3.4.6 Subgroup and sensitivity analyses

Quantitative assessment of heterogeneity revealed substantial levels across primary outcomes. The *I*^2^ statistic was 84.4% for CRP, 87.0% for ALB, and 92.7% for IBDQ. Consequently, a random-effects model was employed for all analyses to incorporate this heterogeneity. Subgroup analyses were performed on the basis of intervention duration (≤6 weeks vs. > 6 weeks). A key finding emerged: the long-term intervention subgroup (>6 weeks) for CRP reduction exhibited moderate heterogeneity (*I*^2^ = 59%, *p* = 0.062). Despite this heterogeneity, the treatment effect remained robust and clinically meaningful, CRP levels were significantly lower in the intervention group than in the control group [MD = −2.56 mg/L, 95% CI (−3.77, −1.35)]. The presence of heterogeneity in longer durations, as opposed to shorter ones, suggests that the effect of dietary interventions on inflammation may not be uniform over time. This could be due to diverging patient adherence, dietary adaptations, or the natural history of IBD over extended periods. In contrast, the analysis of quality of life (IBDQ) showed minimal heterogeneity (*I*^2^ = 1.7%, *p* = 0.384) across both short- and long-term studies, with a consistent positive effect [MD = 0.36, 95% CI (0.08, 0.64)], refer to Supplementary Figures 1, 2. This discrepancy suggests that intervention duration may be a significant methodological factor contributing to the heterogeneity of inflammatory markers. Specifically, subgroup analysis revealed that longer intervention duration (>6 weeks) introduced heterogeneity in CRP outcomes, suggesting that patient compliance, dietary adaptation, or disease natural progression over time may account for variations in anti-inflammatory effects. In contrast, improvements in quality of life (IBDQ) demonstrated consistent and minimal heterogeneity across intervention durations, indicating this outcome is less susceptible to methodological variability related to intervention length.

We further conducted subgroup analyses by disease subtype (CD, UC and IBD). For the CRP outcome, dietary intervention was associated with a significant reduction in UC patients [MD = −1.87 mg/L, 95% CI (−2.86, −0.87)], despite substantial heterogeneity (*I*^2^ = 74.1%). In contrast, no significant effect was observed in CD patients [MD = −0.14, 95% CI (−2.39, 2.11)], with no heterogeneity (*I*^2^ = 0.0%), suggesting a differential anti-inflammatory effect. Detailed results are provided in Supplementary Figures 3, 4. For the IBDQ outcome, a meta-analysis was not feasible for CD due to insufficient data (only one study), underscoring a critical evidence gap. Although a significant improvement was observed in UC [MD = 1.69, 95% CI (0.70 to 2.69)], this result was accompanied by considerable heterogeneity (*I*^2^ = 86.7%). Moreover, analyses for both UC and CD subgroups were ultimately limited by extreme heterogeneity (*I*^2^ > 90%) and confidence intervals that crossed the null value in other included studies, indicating inconsistent effects across the literature. This pervasive heterogeneity likely stems from methodological diversity in how quality of life is influenced and measured, rather than from true differential effects between CD and UC. Consequently, no reliable conclusions can be drawn regarding IBDQ from the current evidence. The divergent findings between CD and UC patients highlight disease subtype as a major source of clinical heterogeneity. These findings highlight the necessity for future trials to be sufficiently powered to analyze CD and UC separately and to employ standardized outcome measures to generate clinically actionable evidence.

Sensitivity analysis via the leave-one-out method demonstrated robust pooled estimates (the MD and 95% CI remained stable upon sequential study exclusion), indicating methodological stability (Supplementary Figure 5). Sequential exclusion of potential outlier studies (19, 22, 28, 31, 37) significantly attenuated heterogeneity. These studies likely contributed to heterogeneity through variations in baseline disease characteristics, concomitant therapies, potential unmeasured confounding factors and other methodological factors.

#### 3.4.7 Adverse reactions

We systematically extracted adverse event (AE) data from all included studies. Among the included RCTs, 5 reported adverse events: the EN group experienced gastrointestinal symptoms (2 cases of abdominal pain/bloating, 2 vomiting, 1 diarrhea) likely related to formula osmolarity or infusion rate (17, 36); the LFD group had one IBD recurrence and one withdrawal due to non-compliance (28). Two other RCTs explicitly reported no clinically adverse reactions (22, 26). Overall, the limited data on adverse events prevent a reliable assessment of the long-term safety of these diets. This important gap must be considered by clinicians weighing the benefits against the risks.

#### 3.4.8 Assessment of publication bias

**Figure 6:**
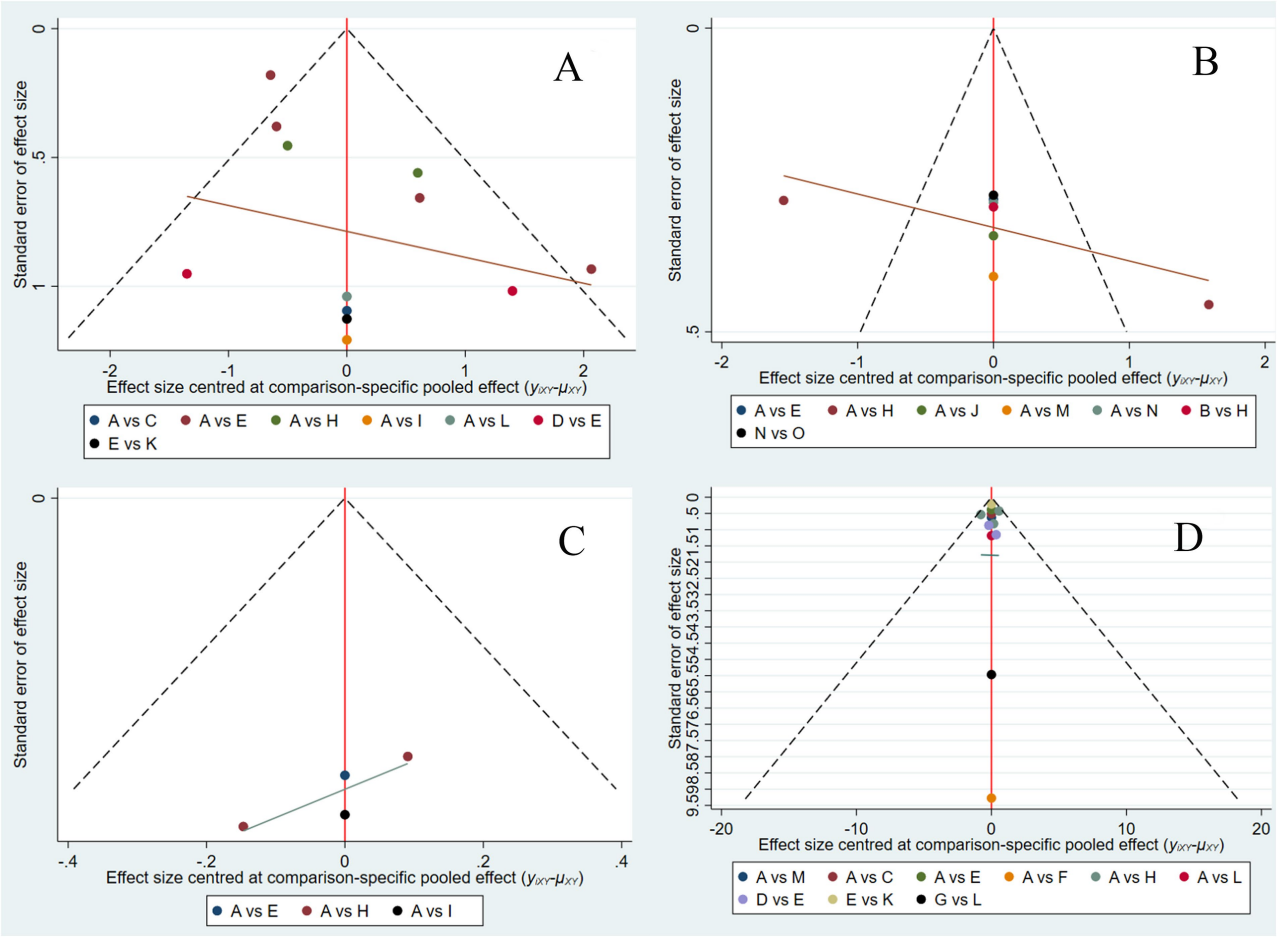
Comparison-correction funnel plot of each outcome indicator. CRP **(A)**, ALB **(B)**, IBDQ **(C)**, and MES **(D)**. A: RD, regular diet; B: HFD, high-FODMAP diet; C: MD, Mediterranean diet; D: LRD, low-residue diet; E: EN, enteral nutrition; F: SCD, specific carbohydrate diet; G: CDED, Crohn’s disease exclusion diet; H: LFD, low-FODMAP diet; I: IgG-ED, IgG-guided exclusion diet; J: HFF, high-fiber food; K: LFD + EN; L: CDED + EN; M: MD + LFD + EN; N: Canada’s Food Guide; O: anti-inflammatory diet.

A comparison-adjusted funnel plot was generated for the 25 included studies, where each data point represents an individual study. Visual inspection revealed that most studies were distributed within the funnel plot symmetry limits and evenly dispersed about the midline, suggesting a low probability of publication bias. However, the observed asymmetry with outliers beyond the funnel limits may indicate potential small-study effects, possibly due to the limited number of studies available for this outcome (Figure 6).

## 4 Discussion

### 4.1 Summary of the main results

Malnutrition is particularly susceptible for IBD patients for multiple mechanisms, including reduced dietary intake, malabsorption, impaired digestion, and medication-related adverse effects. Malnutrition in IBD patients is associated with significantly worse clinical outcomes, including increased disease recurrence, complication rates, hospitalization frequency, prolonged hospital stays, and elevated mortality (38, 39). Appropriate dietary interventions ensure adequate nutrient provision, thereby improving immunocompetence and nutritional status, which are factors that may favor disease progression and clinical outcomes. Evidence suggests that structured dietary modifications can show superior efficacy to pharmacotherapy in select patient subgroups, offering proven effectiveness and established safety in IBD management.

While several previous systematic reviews and meta-analyses have evaluated the efficacy of individual dietary interventions in IBD patients, this is the network meta-analysis that enables a simultaneous comparison and ranking of the relative effectiveness of multiple dietary patterns. We identified 25 eligible studies evaluating 15 distinct dietary interventions in IBD patients. Using network meta-analysis, we assessed multiple outcome measures, including CRP, ALB, the IBDQ, and the MES. The analysis aimed to identify optimal dietary strategies on the basis of comparative effectiveness. Our findings suggest: (1) Effect on inflammatory biomarkers: LFD + EN (SUCRAs: 88.1%) and SCD (SUCRAs: 74.6%) were ranked as the most effective strategies for reducing CRP levels. LFD + EN (SUCRAs: 99.9%) and EN (SUCRAs: 95.6%) were associated with the greatest improvements in serum albumin concentration. (2) Effect on quality of life: MD + LFD + EN (SUCRAs: 70.1%) and HFF (SUCRAs: 66.8%) yielded the most significant benefits in improving IBDQ scores. (3) Effect on endoscopic activity: IgG-ED (SUCRAs: 94%) demonstrated the most pronounced improvement in the MES, ranking superior to all other dietary interventions.

The high SUCRA rankings for interventions like LFD + EN, EN, and IgG-ED should be interpreted in the context of the network meta-analysis methodology. SUCRA synthesizes both direct and indirect evidence, allowing an intervention to rank highly even when direct head-to-head RCTs are scarce, provided the network estimates are consistent and precise. For instance, LFD + EN acts as a common and well-connected comparator within the evidence network. This connectivity, despite the limited number of trials, enhances the reliability of its relative effect estimates through multiple comparison pathways, which is reflected in its high SUCRA value. Similarly, the high ranking of IgG-ED is supported by strong indirect evidence facilitated by common comparators like standard therapy. Furthermore, the use of a random-effects model accounts for heterogeneity and mitigates the risk of overestimating effect sizes from studies with small sample sizes (*n* < 100) by appropriately down-weighting those with greater uncertainty. Crucially, the league table of pairwise comparisons revealed that the differences between top-ranked interventions (SCD, MD + LFD + EN, HFF) were often statistically non-significant. This indicates that while these diets are among the most effective options for their respective outcomes (as shown by high SUCRA), they may not be statistically superior to all alternatives. Therefore, the SUCRA rankings should be viewed as indicating a cluster of promising interventions rather than a definitive hierarchy. Future research should prioritize large, multicenter, randomized head-to-head trials among the top-ranking dietary strategies identified in this analysis (e.g., LFD + EN vs. SCD) to conclusively determine their comparative efficacy and safety.

### 4.2 Comparison with other studies

Systematic reviews and meta-analyses have evidenced that EN is effective for both inducing and maintaining remission in CD, suggesting it can serve as an adjunct to pharmacological therapies (40, 41). A separate systematic review with a meta-analysis demonstrated that LFD could reduce the intestinal digestive and absorptive burdens, thereby alleviating gastrointestinal symptoms (42). These findings support the therapeutic potential of LFD in clinical practice. Our results are consistent with these established effects of dietary interventions. Notably, our study provides novel evidence by comparatively evaluating 15 dietary interventions, including combination therapies, and ranking their efficacy using four original assessment parameters not previously reported in the literature.

### 4.3 Explanation of the research results

#### 4.3.1 CPR/ALB

A universally accepted Minimal Clinically Important Difference (MCID) for CRP in IBD remains elusive due to its variability across populations and disease states. However, the clinical relevance of these reductions is underscored by international consensus, which links a CRP decrease of >5 mg/L or normalization to a level <5 mg/L to endoscopic improvement (43). The reduction achieved by LFD + EN versus LRD (−5.21 mg/L) meets this benchmark, indicating that its anti-inflammatory effect is not only statistically significant but also clinically meaningful. Furthermore, the reductions observed in comparisons with RD, CDED + EN, and LFD (all approximately −4.5 mg/L) approach this threshold of clinical importance. While a universal MCID for albumin is not established, its value as a key marker of nutritional status and disease prognosis is well-documented. Hypoalbuminemia (serum albumin <35 g/L) is a recognized predictor of poor outcomes in IBD, including increased risks of hospitalization, surgery, and postoperative complications (44). The observed increases (3.64–8.73 g/L) are of a magnitude sufficient to correct this deficit, indicating a clinically meaningful improvement in nutritional status.

In terms of CRP and ALB, LFD + EN is the most recommended dietary pattern. However, it is important to note that the observed improvement in systemic inflammatory markers (CRP) and nutritional markers (ALB) is likely not primarily driven by the low-FODMAP component itself, as its main mechanism of action targets functional symptoms. The role of LFD is primarily to alleviate functional gastrointestinal symptoms by modulating osmotic activity and fermentation processes (45). As demonstrated by Ziwei Ni (46), poorly absorbed short-chain carbohydrates increase water volume in the intestinal lumen, accelerating intestinal transit and potentially compromising nutrient absorption in the small intestine. Subsequent microbial fermentation of these substrates in the colon produces gaseous byproducts, contributing to abdominal distension and discomfort. By reducing intake of these fermentable compounds, the LFD component alleviates these physiological processes, thereby improving gut comfort and potentially creating a more favorable environment for nutrient absorption (47). Furthermore, LFD contain specific prebiotics that modulate immune function through multiple mechanisms. These include suppressing the NF-κB proinflammatory signaling pathway, enhancing regulatory T-cell generation, activating the NLRP3 inflammasome, increasing vitamin D receptor expression in intestinal epithelial cells, and attenuating immune hyperreactivity induced by dietary fiber fermentation products (48). Nevertheless, the reduction in inflammatory burden is more plausibly attributed to the effects of enteral nutrition and potential confounding factors, such as concomitant medical therapies and overall improvement in nutritional status. EN formulations provide balanced nutrition while reducing exposure to food additives and macromolecular antigens that may perpetuate immune activation (44). Furthermore, EN enhances intestinal mucosal barrier integrity. In contrast, inflammatory processes increase mucosal permeability through TNF-α-mediated disruption of tight junction proteins in intestinal epithelial cells. Notably, polymer-formulated EN counteracts the effects of TNF-α, restoring tight junction structure and function and improving barrier integrity in intestinal epithelial monolayers (49). In conclusion, the synergistic effect of LFD + EN lies in their complementary mechanisms: EN addresses the core inflammatory and nutritional aspects of IBD through immunomodulation and barrier reinforcement, while LFD provides ancillary benefits by managing coexisting functional gut symptoms prevalent in IBD patients (50). This combination offers a comprehensive dietary approach that targets both pathological inflammation and symptomatic burden in IBD management.

#### 4.3.2 IBDQ

The network meta-analysis did not detect statistically significant differences in IBDQ scores among dietary comparisons. However, it is important to interpret this finding in context. The absence of significance may be explained by several methodological and clinical considerations. First, the IBDQ, though a validated instrument, may have limited sensitivity to detect subtle, yet clinically relevant, improvements in quality of life—particularly in trials with modest sample sizes. Second, the duration of many dietary interventions included in this analysis may have been insufficient to translate physiological changes into meaningful patient-reported outcomes. Dietary modifications often require extended periods to manifest measurable effects on daily functioning and well-being. Finally, considerable heterogeneity was observed across studies regarding patient populations, specific dietary protocols, and adherence levels, which likely reduced the ability to detect consistent treatment effects. Due to the absence of statistical significance, interpretation of the MCID is not feasible. Current evidence is insufficient to determine its clinical relevance, underscoring the need for more consistent, high-quality research in this field.

Based on SUCRA rankings, MD + LFD + EN was identified as the most promising dietary intervention for improving quality of IBDQ. On the basis of the IBDQ scores, MD + LFD + EN showed superior efficacy to the other dietary interventions. MD is characterized by a high intake of plant-based foods, whole grains, fish, and monounsaturated fats, with minimal consumption of red meat, processed foods, and added sugars. The high content of antioxidant compounds (e.g., vitamins A/C, *β*-carotene) and essential minerals in MD likely contributes to its observed anti-inflammatory properties. Multiple prospective cohort studies have demonstrated an inverse association between adherence to the MD and IBD incidence, with a particularly pronounced reduction in the risk of CD. Adherence to the MD in CD patients is associated with significant improvements in quality-of-life metrics, a reduction in clinical disease activity, and decreased fecal calprotectin levels (51, 52). In addition to its anti-inflammatory effects, the MD’s high fiber content aids IBD management through the stimulation of short-chain fatty acid production and the promotion of a beneficial gut microbiota (53, 54). Dietary fiber accelerates intestinal transit, enhances satiety signaling, and fosters colonic fermentation, thereby creating a symbiotic microenvironment that promotes commensal bacterial growth. The combination of MD, LFD, and EN demonstrates superior efficacy in managing IBD symptoms, optimizing CRP and ALB levels, with benefits similar to those of other comprehensive dietary regimens.

#### 4.3.3 MES

The clinical significance in MES is well-established. In ulcerative colitis, achieving endoscopic remission, defined as a MES ≤1, is a key treatment goal (55). The standardized mean differences observed (ranging from SMD 0.64 to 1.07) represent substantial reductions in absolute MES units, confirming the clinical relevance of IgG-ED, LFD, and EN.

The IgG-ED represents a cornerstone of MES. This diet aims to reduce symptoms and inflammation through systematic avoidance of IgG-reactive food antigens. IgG antibodies develop as adaptive immune responses to prolonged dietary antigen exposure, which are distinct from autoimmune responses. Clinical evidence suggests that IgG-ED may benefit IBD patients, as serum IgG titers significantly correlate with disease activity scores in both UC and CD. Gradual elimination of dietary antigens has demonstrated efficacy in reducing intestinal inflammation, likely through decreased immune complex formation (56). According to prior studies, patient compliance remains relatively high. Moreover, individualized plans guided by serological responses to food antigens not only enhance therapy efficacy but may further improve compliance. The clinical adoption of IgG-ED has been facilitated by the widespread application of food-specific IgG testing in specialized laboratories and tertiary hospitals. This approach is expected to gain further traction in clinical practice.

## 5 Limitations and strengths

The strength of this study is that it represents the first NMA to systematically compare and rank the effects of various dietary interventions on CRP, ALB, the IBDQ, and the MES in patients with IBD. This network meta-analysis provides clinically actionable evidence to inform optimal nutritional intervention strategies for IBD patient management. This study has several limitations that warrant consideration. (1) The small sample sizes characterizing the sole studies available for some interventions may compromise the reliability of the associated findings. This is especially true for interventions evaluated in only a single study, making their effect estimates particularly susceptible to the bias and limitations inherent to that primary study, and precluding any robust conclusions. (2) The substantial variability in some trial design—particularly in sample sizes and intervention durations—introduced significant statistical heterogeneity. This heterogeneity could compromise the comparability across studies. (3) The absence of blinding in several trials is a key methodological concern. The lack of participant blinding could influence adherence and co-interventions, while the lack of outcome assessor blinding is particularly critical for subjective patient-reported outcomes (e.g., IBDQ), potentially leading to an overestimation of the treatment effect. Although objective biomarkers (e.g., CRP) are less susceptible, the overall risk of bias remains. (4) The generalizability of our findings may be limited by a linguistic bias, as the inclusion criteria were restricted to Chinese and English studies, excluding relevant non-English literature. (5) Studies reporting multiple outcomes exhibited substantial heterogeneity, potentially due to methodological differences, diverse patient populations, and variations in outcome assessment. (6) The insufficient and inconsistent reporting of adverse events across studies, which prevented a meaningful assessment of the long-term safety and tolerability of the dietary interventions examined.

## 6 Conclusion

This network meta-analysis incorporated 25 randomized controlled trials. Although diverse dietary interventions demonstrated varying degrees of efficacy in alleviating IBD symptoms, LFD + EN was ranked highest for improving inflammatory biomarkers. Furthermore, MD + LFD + EN and IgG-ED among the top-ranked strategies for symptom management. These findings can inform shared decision-making between clinicians and patients regarding evidence-based nonpharmacological therapies tailored to individual characteristics and preferences for IBD symptom management.

Building on our findings, future research should be directed along several specific pathways. First, head-to-head RCTs are strongly recommended to directly compare the top-ranked interventions identified in this analysis, such as the LFD + EN versus MD + LFD + EN. Second, developing international consensus is advised to standardize the implementation, reporting, and adherence monitoring of dietary interventions in IBD trials, which would be crucial for reducing heterogeneity and improving the comparability of future studies. Third, long-term follow-up studies are warranted to evaluate the sustained efficacy, safety, and practicality of these diets in maintaining clinical remission. Finally, future studies should strive to identify predictive biomarkers of treatment response, such as baseline gut microbiota composition or host genetic factors, to ultimately facilitate a personalized nutritional approach for IBD management.

## Data Availability

The original contributions presented in the study are included in the article/Supplementary material, further inquiries can be directed to the corresponding author.

## Glossary

IBD: Inflammatory bowel disease
CRP: C-reactive protein
ALB: Albumin
IBDQ: Inflammatory Bowel Disease Questionnaire
MES: Mayo Endoscopic Score
RD: Regular diet
HFD: High-FODMAP diet
MD: Mediterranean diet
LRD: Low-residue diet
EN: Enteral nutrition
SCD: Specific carbohydrate diet
CDED: Crohn’s disease exclusion diet
LFD: Low-FODMAP diet
IgG-ED: IgG-guided exclusion diet
HFF: High-fiber foods
CFG: Canada’s Food Guide
AID: Anti-inflammatory diet
CD: Crohn’s disease
UC: Ulcerative colitis
ECCO: European Crohn’s and Colitis Organization
CMA: Chinese Medical Association
RCT: Randomized controlled trial
RoB: Risk of bias
SMD: Standardized mean difference
MD: Mean difference
SUCRA: Surface under the cumulative ranking curve
AE: Adverse event
MCID: Minimal Clinically Important Difference
