# Supplementary material for: The efficacy of dietary therapies in modulating inflammatory biomarkers, clinical remission and quality of life in patients with inflammatory bowel disease: a network meta-analysis of 15 interventions
Source: Front Nutr. 2025 Oct 6;12:1668590. doi: 10.3389/fnut.2025.1668590 (PMC12536501; doi:10.3389/fnut.2025.1668590)

Supplementary Material

**Supplementary Table 1.** Search strategy

- 1. CNKI

TKA=('炎症性肠病'+'克罗恩病'+'溃疡性结肠炎') AND TKA=('特定碳水饮食'+'无麸质饮食'+'低FODMAP饮食'+'地中海饮食'+'克罗恩病排除饮食'+'肠内营养') AND FT='随机'

- 1. WanFang Data

主题:("炎症性肠病"or"克罗恩病"or"溃疡性结肠炎") and 主题:("特定碳水饮食"or"无麸质饮食"or"低FODMAP饮食"or"地中海饮食"or"克罗恩病排除饮食"or"肠内营养") 全部:(随机)

- 1. VIP

(M=(炎症性肠病 or 克罗恩病 or 溃疡性结肠炎) or K=(炎症性肠病 or 克罗恩病 or 溃疡性结肠炎)) and (M=(特定碳水饮食 or 无麸质饮食 or 低FODMAP饮食 or 地中海饮食 or 克罗恩病排除饮食 or 肠内营养) or K=(特定碳水饮食 or 无麸质饮食 or 低FODMAP饮食 or 地中海饮食or 克罗恩病排除饮食 or 肠内营养)) and U=随机

- 1. SinoMed

("随机"[全部字段:智能]) AND (((((((("炎性肠疾病"[不加权:扩展]) OR (("结肠炎, 溃疡性"[不加权:扩展])))) OR (("炎性肠疾病"[常用字段:智能] OR "溃疡性结肠炎"[常用字段:智能] OR "克罗恩病"[常用字段:智能]))))) AND (("特定碳水饮食"[常用字段:智能] OR "无麸质饮食"[常用字段:智能] OR "低FODMAP饮食"[常用字段:智能] OR "地中海饮食"[常用字段:智能] OR "克罗恩病排除饮食"[常用字段:智能] OR "肠内营养"[常用字段:智能]))))

- 1. PubMed

(((("Inflammatory Bowel Diseases"[Mesh]) OR (((Inflammatory Bowel Diseases[Title/Abstract]) OR (Diseases, Inflammatory[Title/Abstract])) OR (Inflammatory Bowel Disease[Title/Abstract]))) OR (("Colitis, Ulcerative"[Mesh]) OR ((((Colitis, Ulcerative[Title/Abstract]) OR (Colitis Gravis[Title/Abstract])) OR (Idiopathic Proctocolitis[Title/Abstract])) OR (Inflammatory Bowel Disease, Ulcerative Colitis Type[Title/Abstract])))) OR ((((((((((((Crohn Disease[Title/Abstract]) OR (Crohn's Disease[Title/Abstract])) OR (Crohns Disease[Title/Abstract])) OR (Crohn's Enteritis[Title/Abstract])) OR (Inflammatory Bowel Disease 1[Title/Abstract])) OR (Regional Enteritis[Title/Abstract])) OR (Enteritis, Granulomatous[Title/Abstract])) OR (Granulomatous Enteritis[Title/Abstract])) OR (Enteritis, Regional[Title/Abstract])) OR (Colitis, Granulomatous[Title/Abstract])) OR (Granulomatous Colitis[Title/Abstract])) OR ("Crohn Disease"[Mesh]))) AND (((((((("Diet, Mediterranean"[Mesh]) OR ((((Diet, Mediterranean[Title/Abstract]) OR (Mediterranean Diet[Title/Abstract])) OR (Diets, Mediterranean[Title/Abstract])) OR (Mediterranean Diets[Title/Abstract]))) OR (("Enteral Nutrition"[Mesh]) OR ((((((((((((((((enteral nutrition[Title/Abstract]) OR (Nutrition, Enteral[Title/Abstract])) OR (Tube Feeding[Title/Abstract])) OR (Feeding, Tube[Title/Abstract])) OR (Enteral Feeding[Title/Abstract])) OR (Feeding, Enteral[Title/Abstract])) OR (Gastric Feeding Tubes[Title/Abstract])) OR (Feeding Tube, Gastric[Title/Abstract])) OR (Feeding Tubes, Gastric[Title/Abstract])) OR (Gastric Feeding Tube[Title/Abstract])) OR (Tube, Gastric Feeding[Title/Abstract])) OR (Tubes, Gastric Feeding[Title/Abstract])) OR (Force Feeding[Title/Abstract])) OR (Feeding, Force[Title/Abstract])) OR (Feedings, Force[Title/Abstract])) OR (Force Feedings[Title/Abstract])))) OR (("FODMAP Diet"[Mesh]) OR (((((((((((((((FODMAP Diet[Title/Abstract]) OR (Diet, Low FODMAP[Title/Abstract])) OR (Diets, Low FODMAP[Title/Abstract])) OR (FODMAP Diet, Low[Title/Abstract])) OR (FODMAP Diets, Low[Title/Abstract])) OR (Low FODMAP Diets[Title/Abstract])) OR (Low-FODMAP Diet[Title/Abstract])) OR (Diet, Low-FODMAP[Title/Abstract])) OR (Diets, Low-FODMAP[Title/Abstract])) OR (Low-FODMAP Diets[Title/Abstract])) OR (FODMAP-Containing Diet[Title/Abstract])) OR (Diet, FODMAP-Containing[Title/Abstract])) OR (Diets, FODMAP-Containing[Title/Abstract])) OR (FODMAP Containing Diet[Title/Abstract])) OR (FODMAP-Containing Diets[Title/Abstract])))) OR (("Diet, Gluten-Free"[Mesh]) OR (((((Diet, Gluten Free[Title/Abstract]) OR (Gluten-Free Diet[Title/Abstract])) OR (Diets, Gluten-Free[Title/Abstract])) OR (Gluten Free Diet[Title/Abstract])) OR (Gluten-Free Diets[Title/Abstract])))) OR (specific Carbohydrate Diet[Title/Abstract])) OR (Crohn′s disease exclusion diet[Title/Abstract])) OR (High Fiber Food[Title/Abstract]))

- 1. Web of science

| Search number | Query |
| --- | --- |
| #1 | TS=(Inflammatory Bowel Diseases OR Bowel Diseases, Inflammatory OR Inflammatory Bowel Disease) |
| #2 | TS=(Colitis, Ulcerative OR Colitis Gravis OR Idiopathic Proctocolitis OR Inflammatory Bowel Disease, Ulcerative Colitis Type) |
| #3 | TS=(Crohn Disease OR Crohn's Disease OR Crohns Disease OR Crohn's Enteritis OR Inflammatory Bowel Disease 1 OR Regional Enteritis OR Enteritis, Granulomatous OR Granulomatous Enteritis OR Enteritis, Regional OR Colitis, Granulomatous OR Granulomatous Colitis) |
| #4 | #1 OR #2 OR #3 |
| #5 | TS=(specific Carbohydrate Diet OR gluten free diet OR Low FODMAPs Diet OR Mediterranean Diet OR Anti-inflammatory diet OR Crohn′s disease exclusion diet OR enteral nutrition) |
| #6 | #4 AND #5 |

- 1. cochrane library

| Search number | Query |
| --- | --- |
| #1 | MeSH descriptor: [Inflammatory Bowel Diseases] explode all trees |
| #2 | Inflammatory Bowel Diseases OR Low FODMAPs Diet OR Inflammatory Bowel Disease |
| #3 | MeSH descriptor: [Colitis, Ulcerative] explode all trees |
| #4 | Idiopathic Proctocolitis OR Ulcerative Colitis Type OR Ulcerative Colitis OR Colitis Gravis |
| #5 | MeSH descriptor: [Crohn Disease] explode all trees |
| #6 | Ileocolitis OR Regional Ileitides OR Ileitis, Regional OR Terminal Ileitis OR Regional Ileitis OR Ileitis, Terminal OR Enteritis, Granulomatous OR Enteritis, Regional OR Granulomatous Enteritis; Crohn's Disease OR Crohn's Enteritis OR Regional Enteritis OR Crohns Disease OR Granulomatous Colitis OR Colitis, Granulomatous |
| #7 | #1 OR #2 OR #3 OR #4 OR #5 OR #6 |
| #8 | MeSH descriptor: [Diet,Gluten-Free] explode all trees |
| #9 | MeSH descriptor: [[Diet, Mediterranean](file:///C:\\Users\\Lenovo\\Desktop\\魏珂禧中医外科学22023757\\必发核心班\\饮食\\文章\\Supplementary%20appendix%201.docx#0)] explode all trees |
| #10 | MeSH descriptor: [[Enteral Nutrition](file:///C:\\Users\\Lenovo\\Desktop\\魏珂禧中医外科学22023757\\必发核心班\\饮食\\文章\\Supplementary%20appendix%201.docx#0)] explode all trees |
| #11 | specific Carbohydrate Diet OR Gluten-Free Diets OR Diet, Gluten Free OR Gluten Free Diet OR Gluten-Free Diet OR Diets, Gluten-Free OR Low FODMAPs Diet OR Mediterranean Diet OR Diets, Mediterranean OR Mediterranean Diets OR Anti-inflammatory diet OR Crohn′s disease exclusion diet OR Feeding Tube, Gastric OR Gastric Feeding Tubes OR Feeding Tubes, Gastric OR Tubes, Gastric Feeding OR Tube, Gastric Feeding OR Gastric Feeding Tube OR Nutrition, Enteral OR Force Feedings OR Feeding, Force OR Force Feeding OR Feedings, Force OR Tube Feeding OR Feeding, Tube OR Enteral Feeding OR Feeding, Enteral |
| #12 | #8 OR #9 OR #10 OR #11 |
| #13 | #7 AND #12 |

- 1. Embace

| Search number | Query |
| --- | --- |
| #23 | #7 AND #22 |
| #22 | #8 OR #9 OR #10 OR #11 OR #12 OR #13 OR #14 OR #15 OR #16 OR #17 OR #18 OR #19 OR #20 OR #21 |
| #21 | 'enteral feeding':ab,ti OR 'enteric feeding':ab,ti OR 'enteral nutrition':ab,ti OR 'enteric nutrition':ab,ti OR 'feeding, enteric':ab,ti OR feeding,intragastric:ab,ti OR 'intestinal feeding':ab,ti OR 'intragastric feeding intraintestinal feeding':ab,ti OR 'tube feeding':ab,ti |
| #20 | 'enteric feeding'/exp |
| #19 | cded:ab,ti AND 'crohn`s disease exclusion diet':ab,ti OR 'crohn disease exclusion diet':ab,ti OR 'crohn`s disease exclusion diet':ab,ti |
| #18 | 'crohn disease exclusion diet'/exp |
| #17 | 'anti inflammatory diet':ab,ti |
| #16 | 'anti inflammatory diet'/exp |
| #15 | 'diet, mediterranean':ab,ti OR meddiet:ab,ti OR 'mediterranean diet':ab,ti |
| #14 | 'mediterranean diet'/exp |
| #13 | 'low fodmaps diet':ab,ti OR 'low fodmap diet':ab,ti OR 'fodmap-restricted diet':ab,ti OR 'fodmap-reduced diet':ab,ti OR 'fodmap-restrictive diet':ab,ti OR 'fodmaps-restricted diet':ab,ti OR ('low fermentable oligo-, di-, mono-saccharides':ab,ti AND 'polyol diet':ab,ti) OR 'fodmap elimination diet':ab,ti OR ('low fermentable oligo-, di-, monosaccharides':ab,ti AND 'polyols diet':ab,ti) OR ('low fermentable oligosaccharides, disaccharides, monosaccharides':ab,ti AND 'polyols diet':ab,ti) OR 'low fodmap`s diet':ab,ti OR 'reduced fodmap diet':ab,ti OR 'restricted fodmap diet':ab,ti OR 'restricted fodmaps diet':ab,ti |
| #12 | 'low fodmap diet'/exp |
| #11 | 'diet,gluten free':ab,ti OR 'gluten-free diet':ab,ti OR 'gluten free diet':ab,ti |
| #10 | 'gluten free diet'/exp |
| #9 | 'specific carbohydrate diet':ab,ti |
| #8 | 'specific carbohydrate diet'/exp |
| #7 | #1 OR #2 OR #3 OR #4 OR #5 OR #6 |
| #6 | 'cleron disease':ab,ti OR 'crohns disease':ab,ti OR 'crohn`s disease':ab,ti OR 'enteritis regionalis':ab,ti OR 'intestinal tract, regional enteritis':ab,ti OR 'morbus crohn':ab,ti OR 'crohn disease':ab,ti OR 'colon, chronic ulceration':ab,ti OR 'regional enteritis':ab,ti OR 'regional enterocolitis':ab,ti |
| #5 | 'chronic ulcerative colitis':ab,ti OR 'ulcerative colitis':ab,ti OR 'colitis ulcerosa':ab,ti OR 'colitis ulcerativa':ab,ti OR 'colitis ulcerosa chronica':ab,ti OR 'colitis, mucosal':ab,ti OR 'colitis, ulcerative':ab,ti OR 'colitis, ulcerous':ab,ti OR 'colon, chronic ulceration':ab,ti OR 'histiocytic ulcerative colitis':ab,ti OR 'mucosal colitis':ab,ti OR 'ulcerative colorectitis':ab,ti OR 'ulcerative proctocolitis':ab,ti OR 'ulcerous colitis':ab,ti OR 'ulcerative procto colitis':ab,ti |
| #4 | 'inflammatory bowel diseases':ab,ti OR 'inflammatory bowel disease':ab,ti |
| #3 | 'crohn disease'/exp |
| #2 | 'ulcerative colitis'/exp |
| #1 | 'inflammatory bowel disease'/exp |

For three-arm trials, the two intervention groups were pooled using the following calculations: For Group A (sample size=N1, mean=M1, SD=SD1) and Group B (sample size=N2, mean=M2, SD=SD2), the combined sample size was calculated as N=N1+N2, with weighted mean M=(N1×M1+N2×M2)/(N1+N2). The pooled standard deviation was derived using the following formula:


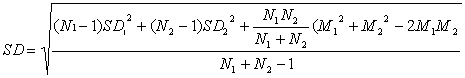

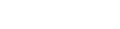


**Supplementary Table 2 Description of Dietary Interventions**

| **Dietary Interventions** | **Description** |
| --- | --- |
| HFD: high-FODMAP diet. | A diet high in Fermentable Oligosaccharides, Disaccharides, Monosaccharides, and Polyols (FODMAPs)[1]. |
| MD: Mediterranean diet. | A dietary pattern inspired by the traditional eating habits of countries bordering the Mediterranean Sea. It emphasizes high consumption of fruits, vegetables, whole grains, legumes, nuts, and olive oil; moderate intake of fish and poultry; and low intake of red meat, processed foods, and sweets. It is rich in fiber, antioxidants, and anti-inflammatory compounds[2]. |
| LRD: low-residue diet. | A diet designed to reduce the frequency and volume of stools by limiting foods that contribute to residue, specifically dietary fiber and other poorly digestible materials. It typically includes refined grains, well-cooked vegetables without skins or seeds, limited fruits, and lean meats[3]. |
| EN: enteral nutrition. | A liquid-only diet consisting of formulated nutritionally complete formulas that are either ingested orally (sips) or administered via a feeding tube (nasogastric or gastrostomy)[4]. |
| SCD: specific carbohydrate diet. | A strict, grain-free and lactose-free diet that eliminates complex carbohydrates (disaccharides and polysaccharides) believed to be poorly absorbed and to feed pro-inflammatory gut bacteria. It allows only specific, well-absorbed monosaccharides from fruits, honey, certain vegetables, nuts, and meats[5]. |
| CDED: Crohn’s disease exclusion diet. | A whole-food-based diet designed to reduce exposure to dietary components that may adversely affect the gut microbiome and intestinal barrier. It is often combined with Partial Enteral Nutrition (PEN). The diet excludes emulsifiers, maltodextrin, carrageenan, and other additives, and limits certain foods like processed meats, dairy, and gluten-rich grains[6]. |
| LFD: low-FODMAP diet. | A diet that limits certain types of carbohydrates (called FODMAPs) found in many common foods like wheat, onions, milk, and some fruits[1]. |
| IgG-ED: IgG-guided exclusion diet. | An elimination diet based on the measurement of food-specific IgG antibodies in the patient's blood. Foods that show elevated antibody levels are removed from the diet for a period, with the hypothesis that this may reduce immune-mediated food sensitivities and improve symptoms[7]. |
| HFF: High-fiber foods. | An intervention focused on increasing the intake of dietary fiber from sources like fruits, vegetables, and whole grains. In the context of IBD, this is typically studied during remission, as fiber can be beneficial for the gut microbiome (acting as a prebiotic) but may be poorly tolerated during active inflammation[8]. |
| CFG: Canada's food guide. | A general population guide to healthy eating published by the Canadian government. It promotes a pattern of eating that encourages plenty of vegetables and fruits, whole grains, and protein foods (with an emphasis on plant-based sources), while limiting highly processed foods[9]. |
| AID: anti-inflammatory diet. | A general term for dietary patterns designed to reduce chronic systemic inflammation. While not standardized, it typically shares features with the Mediterranean diet, emphasizing omega-3 fatty acids (e.g., from fish), antioxidants (e.g., from berries, leafy greens), and whole foods while avoiding processed foods, refined sugars, and unhealthy fats[10]. |

**Supplementary material**

1. Gibson PR, Shepherd SJ: **Evidence-based dietary management of functional gastrointestinal symptoms: The FODMAP approach.** *J Gastroenterol Hepatol* 2010, **25:**252-258.

2. Martínez-González MA, Salas-Salvadó J, Estruch R, Corella D, Fitó M, Ros E: **Benefits of the Mediterranean Diet: Insights From the PREDIMED Study.** *Prog Cardiovasc Dis* 2015, **58:**50-60.

3. Hou JK, Lee D, Lewis J: **Diet and inflammatory bowel disease: review of patient-targeted recommendations.** *Clin Gastroenterol Hepatol* 2014, **12:**1592-1600.

4. van Rheenen PF, Aloi M, Assa A, Bronsky J, Escher JC, Fagerberg UL, Gasparetto M, Gerasimidis K, Griffiths A, Henderson P, et al: **The Medical Management of Paediatric Crohn's Disease: an ECCO-ESPGHAN Guideline Update.** *J Crohns Colitis* 2021, **15**.

5. Suskind DL, Cohen SA, Brittnacher MJ, Wahbeh G, Lee D, Shaffer ML, Braly K, Hayden HS, Klein J, Gold B, et al: **Clinical and Fecal Microbial Changes With Diet Therapy in Active Inflammatory Bowel Disease.** *J Clin Gastroenterol* 2018, **52:**155-163.

6. Levine A, Wine E, Assa A, Sigall Boneh R, Shaoul R, Kori M, Cohen S, Peleg S, Shamaly H, On A, et al: **Crohn's Disease Exclusion Diet Plus Partial Enteral Nutrition Induces Sustained Remission in a Randomized Controlled Trial.** *Gastroenterology* 2019, **157:**440-450.e448.

7. Aydinlar EI, Dikmen PY, Tiftikci A, Saruc M, Aksu M, Gunsoy HG, Tozun N: **IgG-based elimination diet in migraine plus irritable bowel syndrome.** *Headache* 2013, **53:**514-525.

8. Lewis JD, Abreu MT: **Diet as a Trigger or Therapy for Inflammatory Bowel Diseases.** *Gastroenterology* 2017, **152:**398-414.e396.

9. Levine A, Rhodes JM, Lindsay JO, Abreu MT, Kamm MA, Gibson PR, Gasche C, Silverberg MS, Mahadevan U, Boneh RS, et al: **Dietary Guidance From the International Organization for the Study of Inflammatory Bowel Diseases.** *Clin Gastroenterol Hepatol* 2020, **18:**1381-1392.

10. Jeznach-Steinhagen A, Ostrowska J, Czerwonogrodzka-Senczyna A, Boniecka I, Gronostajska W: **[Dietetary recommendation for non-alcoholic fatty liver disease].** *Pol Merkur Lekarski* 2017, **43:**281-286.

Supplementary Figure 1A. Subgroup forest map of intervention time for CRP


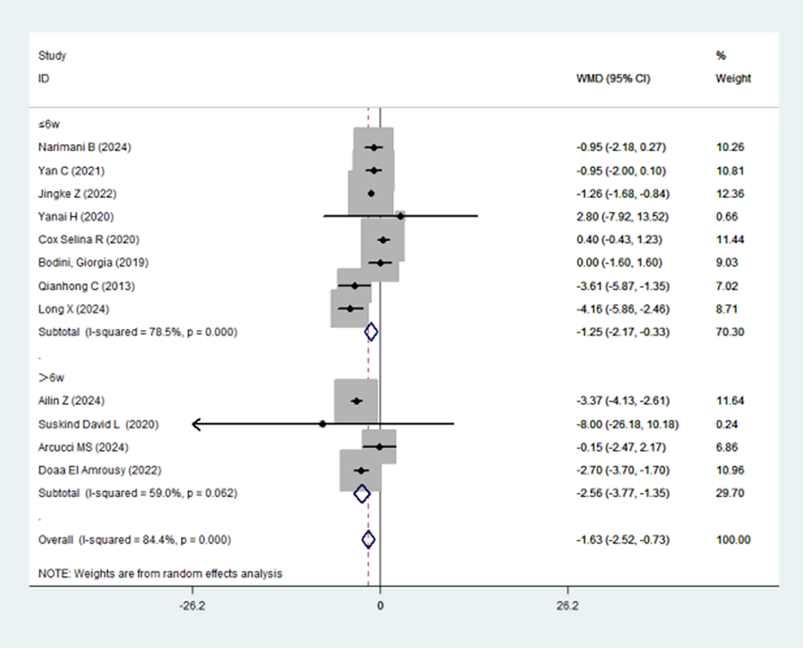


Supplementary Figure 1B. Subgroup forest map of intervention time for ALB


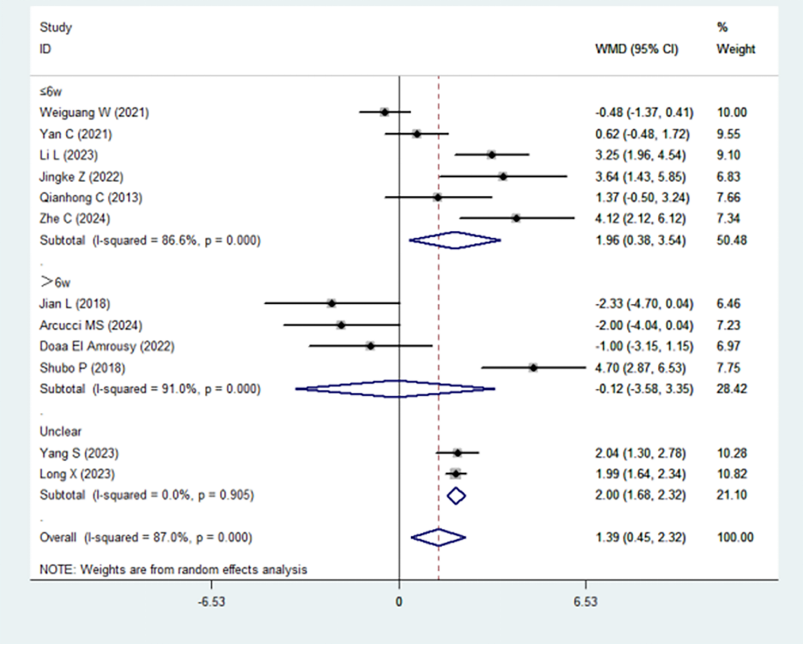


Supplementary Figure 1C. Subgroup forest map of intervention time for IBDQ


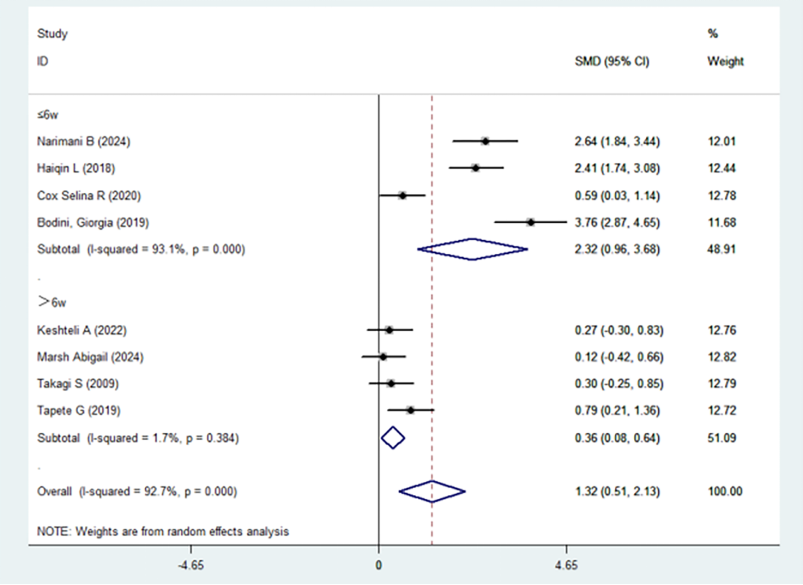


Supplementary Figure 2A. A meta-regression analysis was conducted of intervention time for CRP


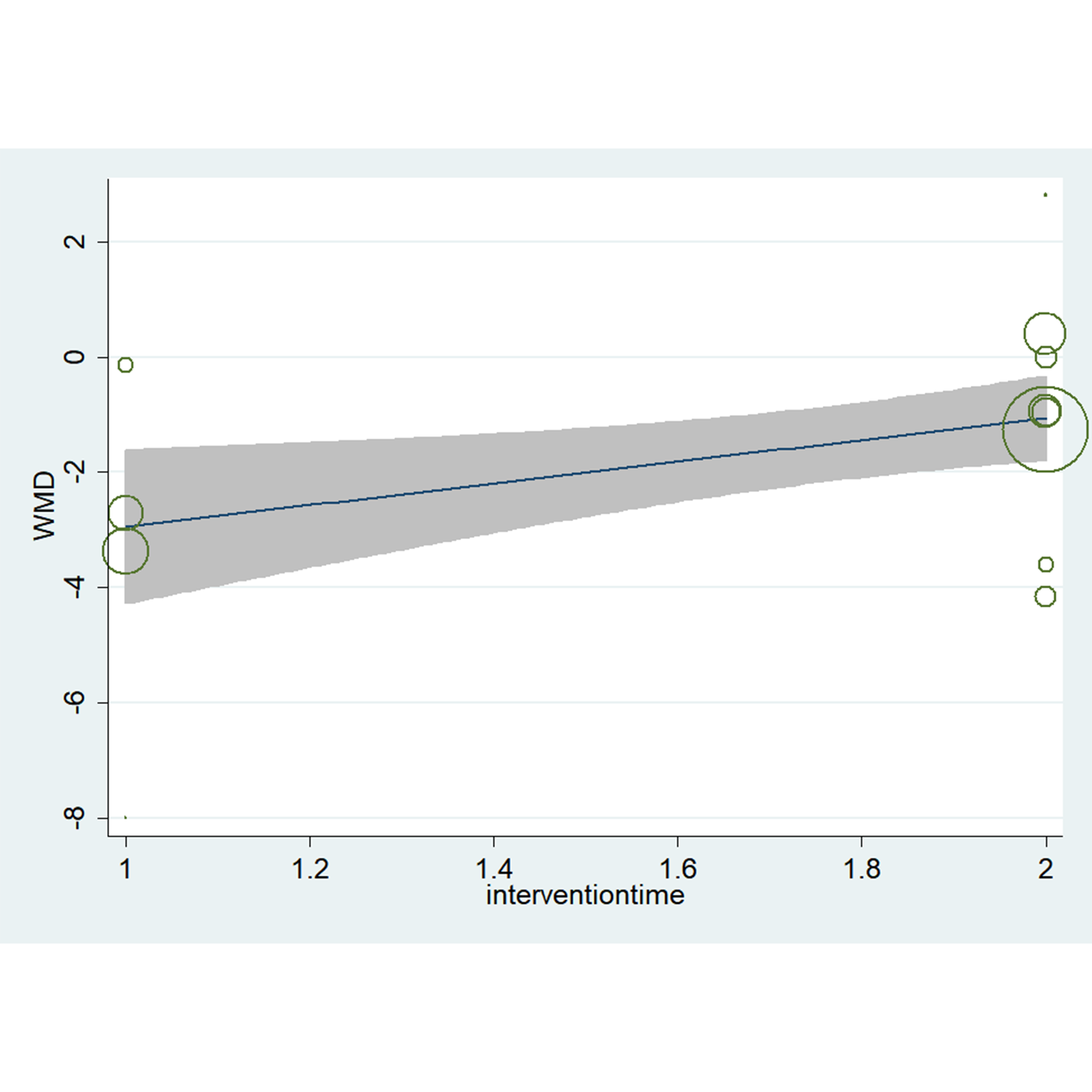


Supplementary Figure 2B. A meta-regression analysis was conducted of intervention time for ALB


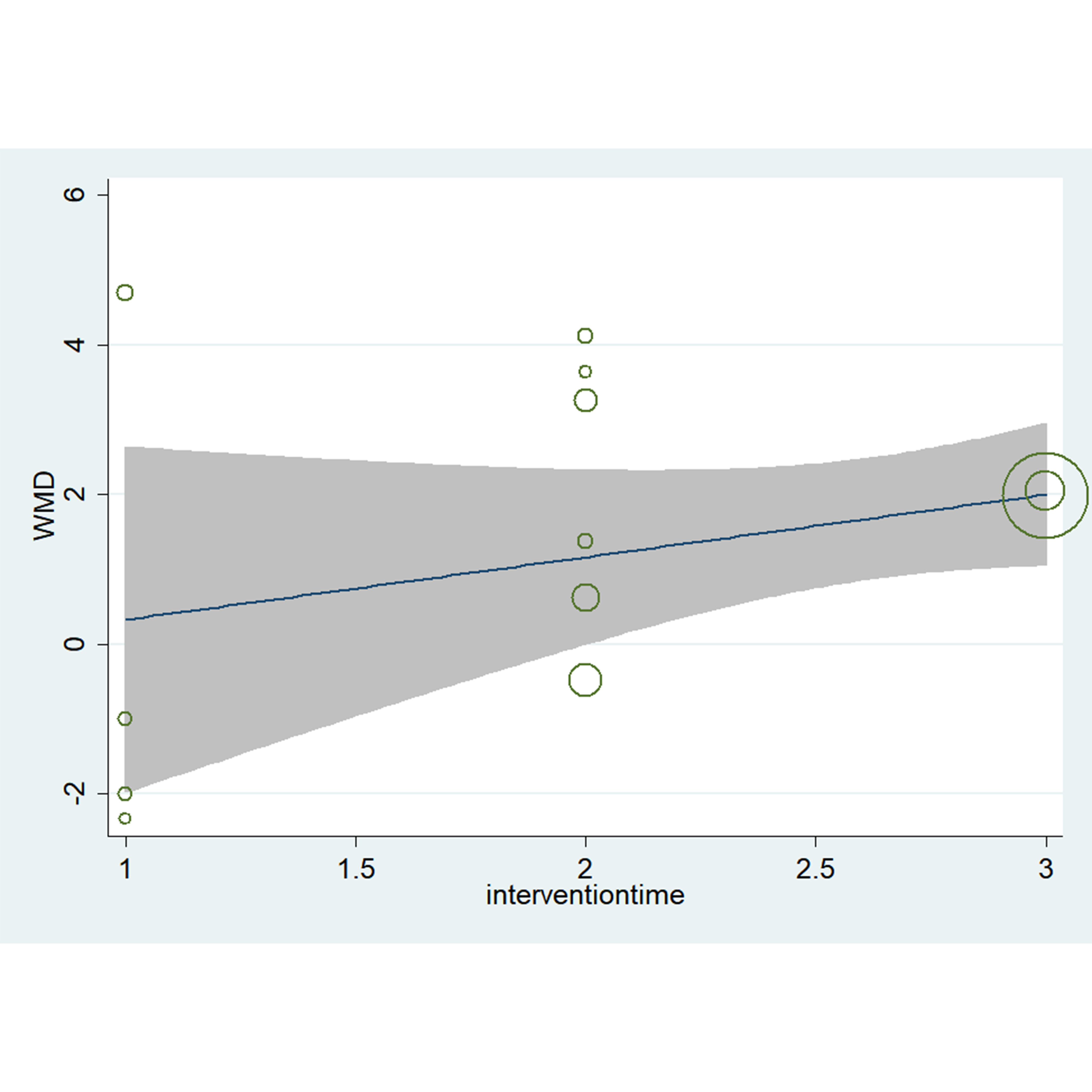


Supplementary Figure 2C. A meta-regression analysis was conducted of intervention time for IBDQ


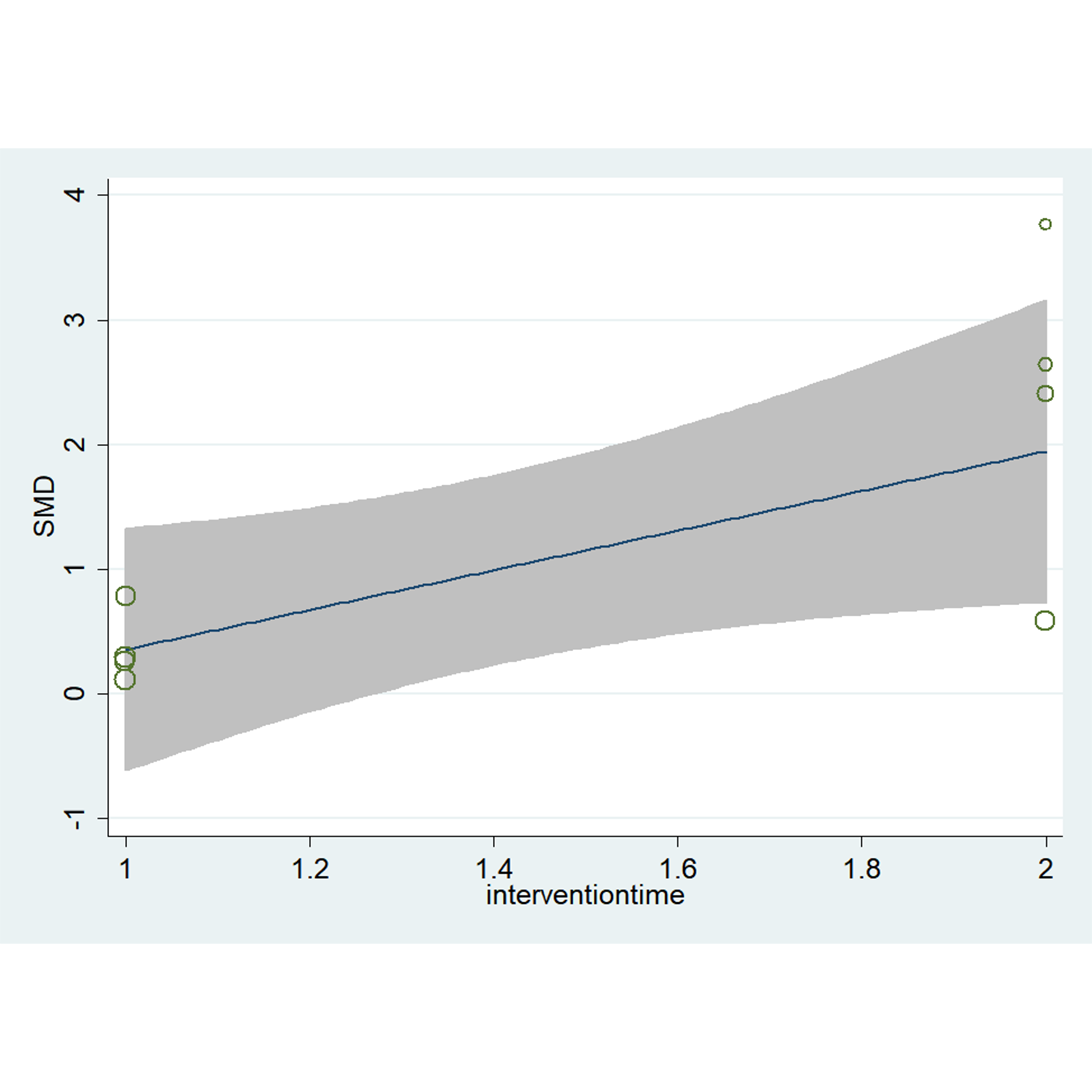


Supplementary Figure 3A. Subgroup analysis of disease types for CRP


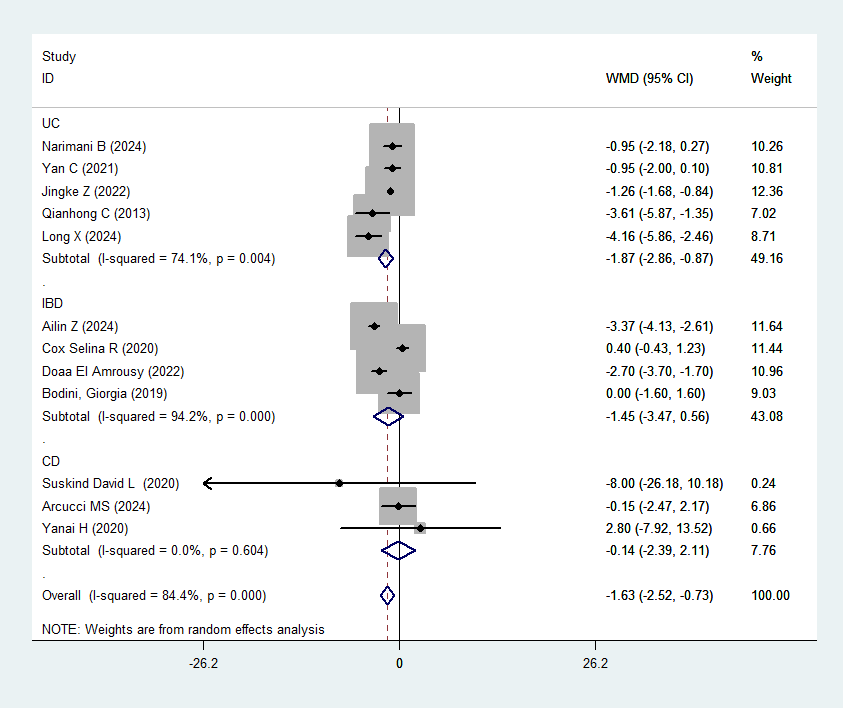


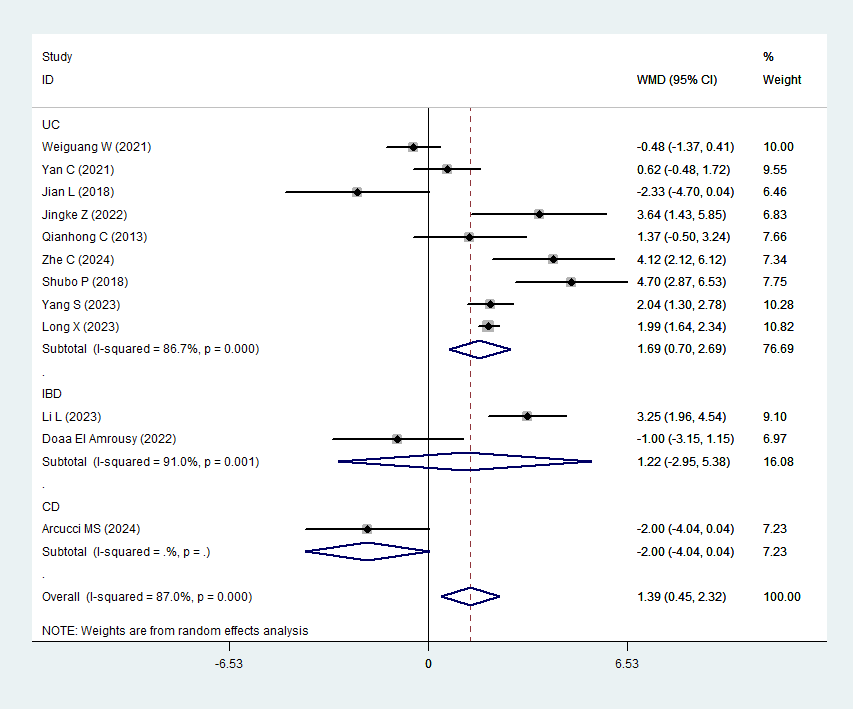
Supplementary Figure 3B. Subgroup analysis of disease types for ALB


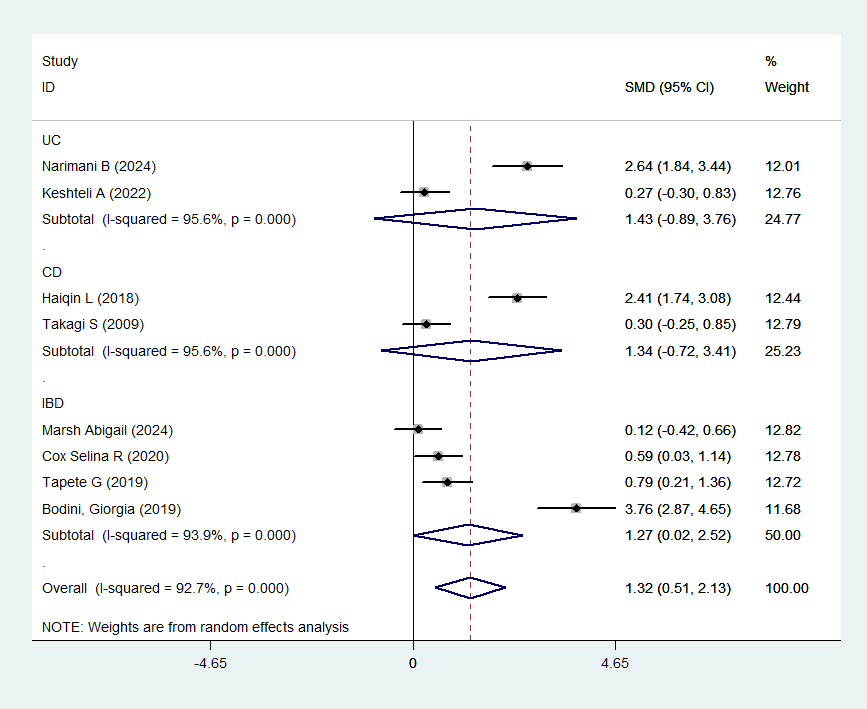
Supplementary Figure 3C. Subgroup analysis of disease types for IBDQ

Supplementary Figure 4A. A meta-regression analysis was conducted of disease types for CRP


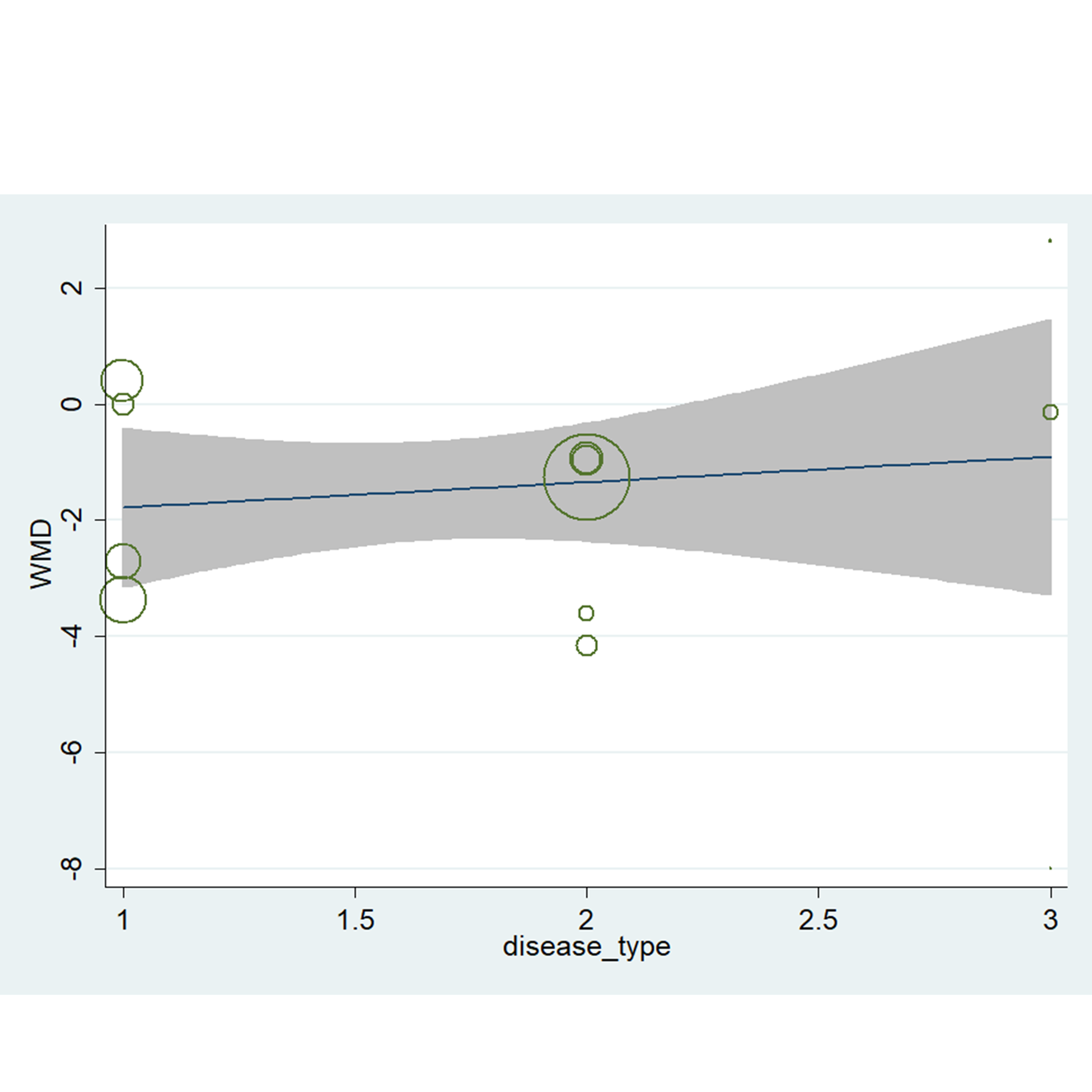


Supplementary Figure 4B. A meta-regression analysis was conducted of intervention time for ALB


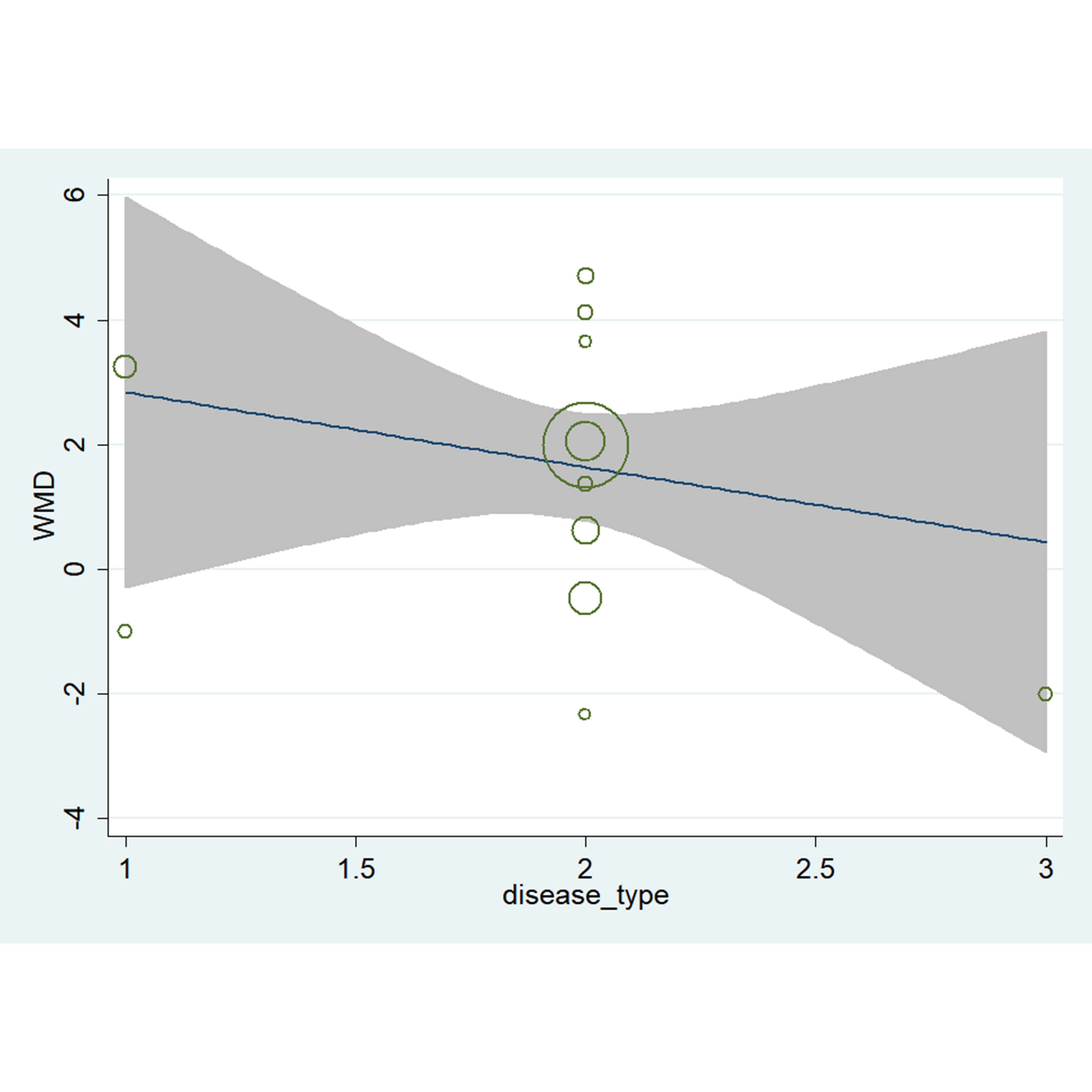


Supplementary Figure 4C. A meta-regression analysis was conducted of intervention time for IBDQ


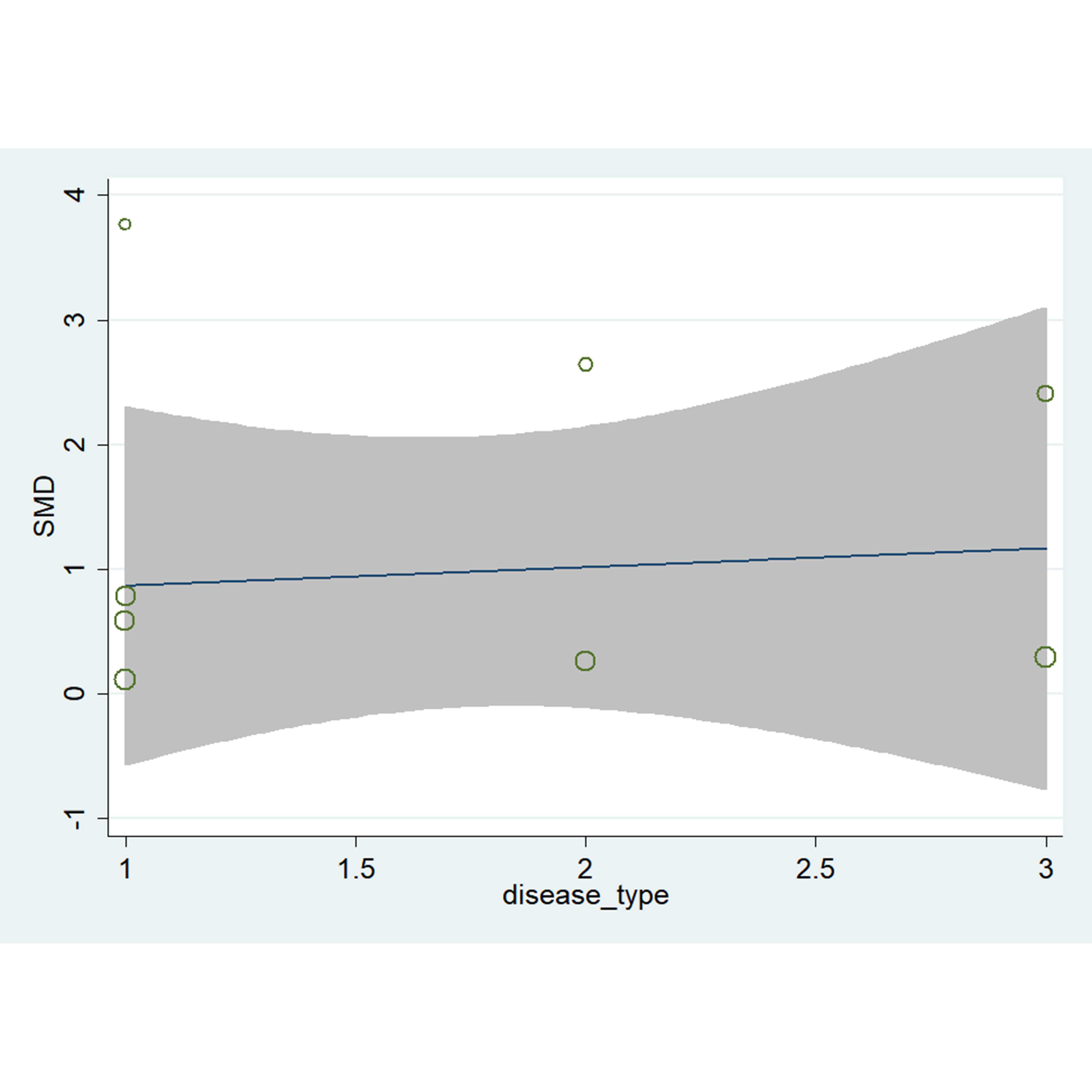


Supplementary Figure 5A. Sensitivity analysis of CPR


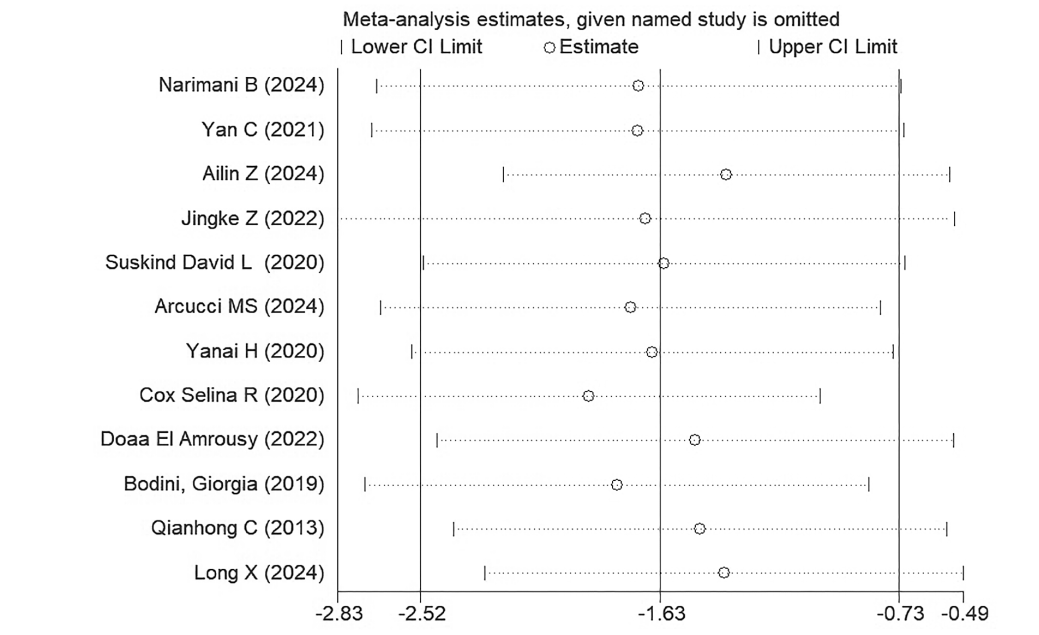


Supplementary Figure 5B. Sensitivity analysis of ALB


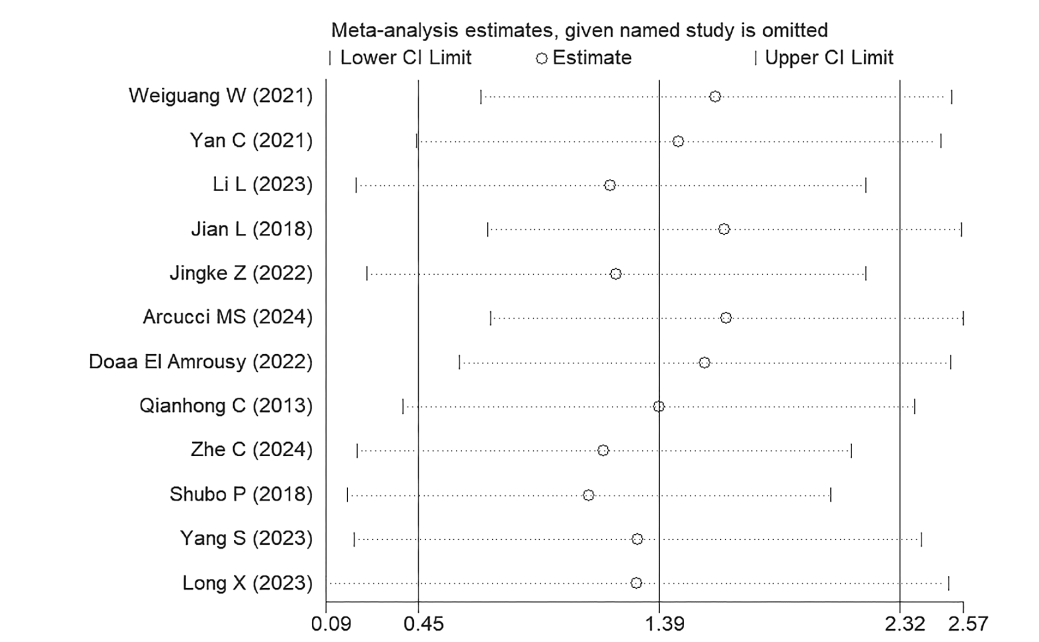


Supplementary Figure 5C. Sensitivity analysis of IBDQ


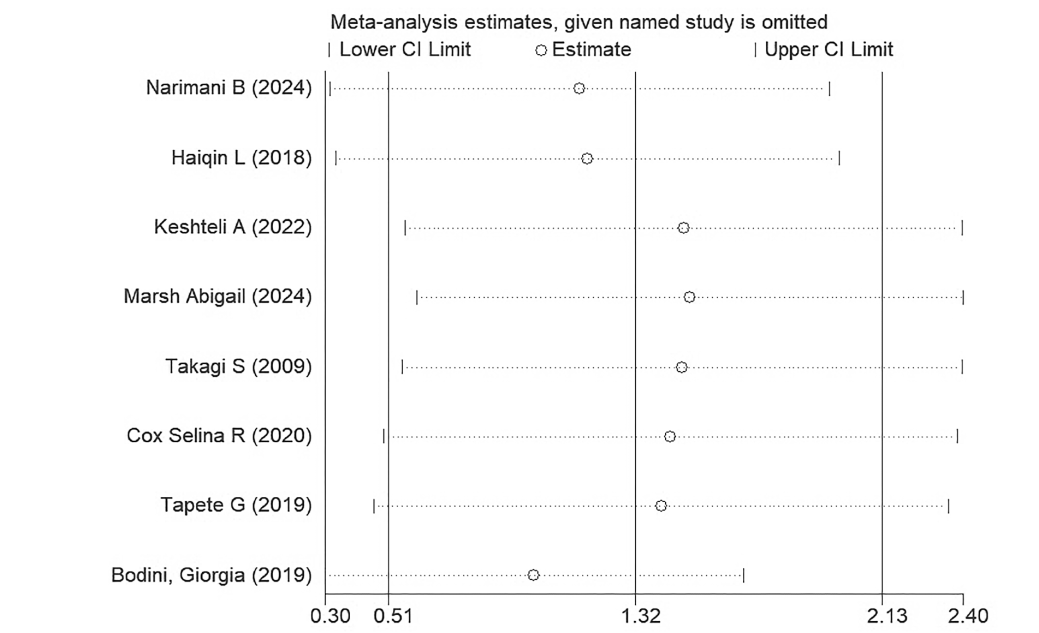

Supplement: Supplementary file 1 [file Table_1.DOCX]
